# Functional CFTR may be required for *Prevotella melaninogenica* regulation of epithelial cell defense against *Staphylococcus aureus*

**DOI:** 10.1016/j.jcf.2025.11.002

**Published:** 2025-11-05

**Authors:** Maksym Goryachok, Ana Fairbanks-Mahnke, Sam Fulte, Emily Tamkin, Arianna McCarty, Eric D. Larson, Paul J. Planet, Sarah E. Clark

**Affiliations:** a University of Colorado School of Medicine, Department of Otolaryngology – Head & Neck Surgery, Aurora, CO, USA; b University of Pennsylvania, School of Dental Medicine, Department of Basic and Translational Science, Philadelphia, PA, USA; c Children’s Hospital of Philadelphia, Perelman School of Medicine, University of Pennsylvania, Department of Pediatrics, Philadelphia, PA, USA

**Keywords:** Cystic fibrosis, CFBE41o-cells, CFTR modulators, Bacterial infection, Prevotella, Staphylococcus aureus

## Abstract

**Background:**

*Prevotella melaninogenica* is enriched in the lungs of people with cystic fibrosis (pwCF), yet its functional impact on respiratory tract homeostasis remains incompletely understood. Prior studies identified immune modulatory effects following lung exposure to *Prevotella*, but the relevance of these findings for CF infections is unknown.

**Methods:**

The impact of *P. melaninogenica* on infection with the CF pathogen *Staphylococcus aureus* was evaluated using a mouse lung infection model and by measuring *S. aureus* adherence to human respiratory tract cystic fibrosis transmembrane conductance regulator (CFTR) mutant and isogenic wild-type (WT)-corrected CFBE41o-epithelial cells. Epithelial cytokine/chemokine secretion and RNA-sequencing were performed to compare *P. melaninogenica*-induced signaling programs in WT-corrected versus CFTR mutant cells.

**Results:**

*P. melaninogenica* significantly reduced *S. aureus* lung infection, associated with elevated *S. aureus* killing by lung neutrophils and impaired *S. aureus* adherence to epithelial cells. Live or killed *P. melaninogenica* were sufficient to mediate these effects, which were dependent on TLR2. *P. melaninogenica* impairment of *S. aureus* adherence required functional CFTR, as this effect was lost in CFTR mutant cells but restored by CFTR modulators. RNA-sequencing identified several antibacterial defense pathways selectively upregulated by *P. melaninogenica* in WT corrected epithelial cells, correlating with higher IL-8 and IL-6 cytokine production.

**Conclusions:**

*P. melaninogenica* enhanced neutrophil and epithelial defense against *S. aureus*, but the benefits of epithelial cell regulation by *P. melaninogenica* were lost with CFTR dysfunction. CFTR modulators rescued *P. melaninogenica* responsiveness in epithelial cells, highlighting the potential for synergistic effects of hostmicrobiome interactions and CFTR targeted therapies.

## 1. Introduction

Advances in cystic fibrosis (CF) therapy over the last decade have substantially improved lung function and reduced pulmonary exacerbations, particularly for people with CF (pwCF) treated with highly effective cystic fibrosis transmembrane conductance regulator (CFTR) modulator therapy (HEMT) [1]. The triple CFTR modulator elexacaftor/tezacaftor/ivacaftor, approved by the Food and Drug Administration in 2019, works by restoring CFTR protein function [2]. HEMT has significantly modified the pulmonary landscape in people with CF (pwCF), including the CF lung microbiome [3]. Alongside improvements in mucociliary clearance, lung microbiome diversity is increased following HEMT, resulting in a higher abundance of non-pathogenic bacteria including oral commensal anaerobes [4-6]. Greater lung microbiome diversity correlates with better lung function in pwCF [5,7], but the mechanisms contributing to this relationship are unclear.

Specific components of the microbiome, including bacteria inhabiting the upper respiratory tract, improve baseline defense against lung infection. In people without CF, greater respiratory tract microbiome diversity and oral commensal anaerobe abundance both correlate with reduced pneumonia mortality [8-10]. *Prevotella* are Gram-negative obligate anaerobes which colonize the oral cavity, are frequently aspirated to the lower airway, and are associated with healthy lung function [11-13]. *Prevotella* are reduced in the context of chronic lung diseases including asthma and chronic obstructive pulmonary disease (COPD) [14,15], and *Prevotella* abundance negatively correlates with bacterial pneumonia from *Streptococcus pneumoniae* and other pathogens [16-19]. In prior work, we recapitulated the negative correlation between *Prevotella melaninogenica* and pneumococcal pneumonia in a mouse infection model, where lung exposure to *P. melaninogenica* improved innate immune mediated clearance of *S. pneumoniae* [20]. However, this effect was species-specific, as *Prevotella intermedia*, which is more frequently associated with periodontal disease [21], failed to protect against *S. pneumoniae* infection and was shown by others to have toxic effects on human respiratory tract epithelial cells [22]. *P. melaninogenica* may itself not always have beneficial effects, as it was shown to worsen tissue damage during Bleomycin-induced pulmonary fibrosis [23]. These findings suggest that *Prevotella* impact pulmonary homeostasis and antibacterial defense, with species and context-specific outcomes.

In pwCF, the impact of *Prevotella* on lung health is poorly understood. *Prevotella* are a core member of the CF microbiome [24-26], and *Prevotella* abundance correlates with improved lung function measured by FEV_1_ or lung clearance index (LCI) in several studies [4,7,25,27-29], including quantitative bacterial culture [28]. In vitro studies using CFTR mutant bronchial epithelial cells (CFBE41o-) have demonstrated that *Prevotella* species including *Prevotella histicola* and *Prevotella nigrescens* activate CFTR mutant epithelial cells and alter the epithelial response to the CF pathogen *Pseudomonas aeruginosa* [30,31]. CF pathogens including *Pseudomonas aeruginosa* and *Staphylococcus aureus* remain an important concern in pwCF, as persistent lung infections significantly alter quality of life and reduce lung function, ultimately contributing to pulmonary failure [3]. *S. aureus* is one of the earliest CF pathogens to be acquired in pwCF, and chronic *S. aureus* infection correlates with reduced lung function [32-34].

While the direct physiological impacts of CFTR mutation are well characterized, less is known regarding the consequences of CFTR mutation on the epithelial response to the non-pathogenic bacteria commonly inhabiting the CF lungs, and whether this helps or hinders infection control. In this study we address the impact of *P. melaninogenica* on epithelial activation and defense against the CF pathogen *S. aureus*. Using isogenic CFTR mutant and wild-type epithelial cells, we identify critical role for functional CFTR in *P. melaninogenica*-induced epithelial cell activation and defense against *S. aureus* infection.

## 2. Results

### 2.1. P. melaninogenica improves neutrophil-mediated defense against S. aureus lung infection

**Fig. 1.**
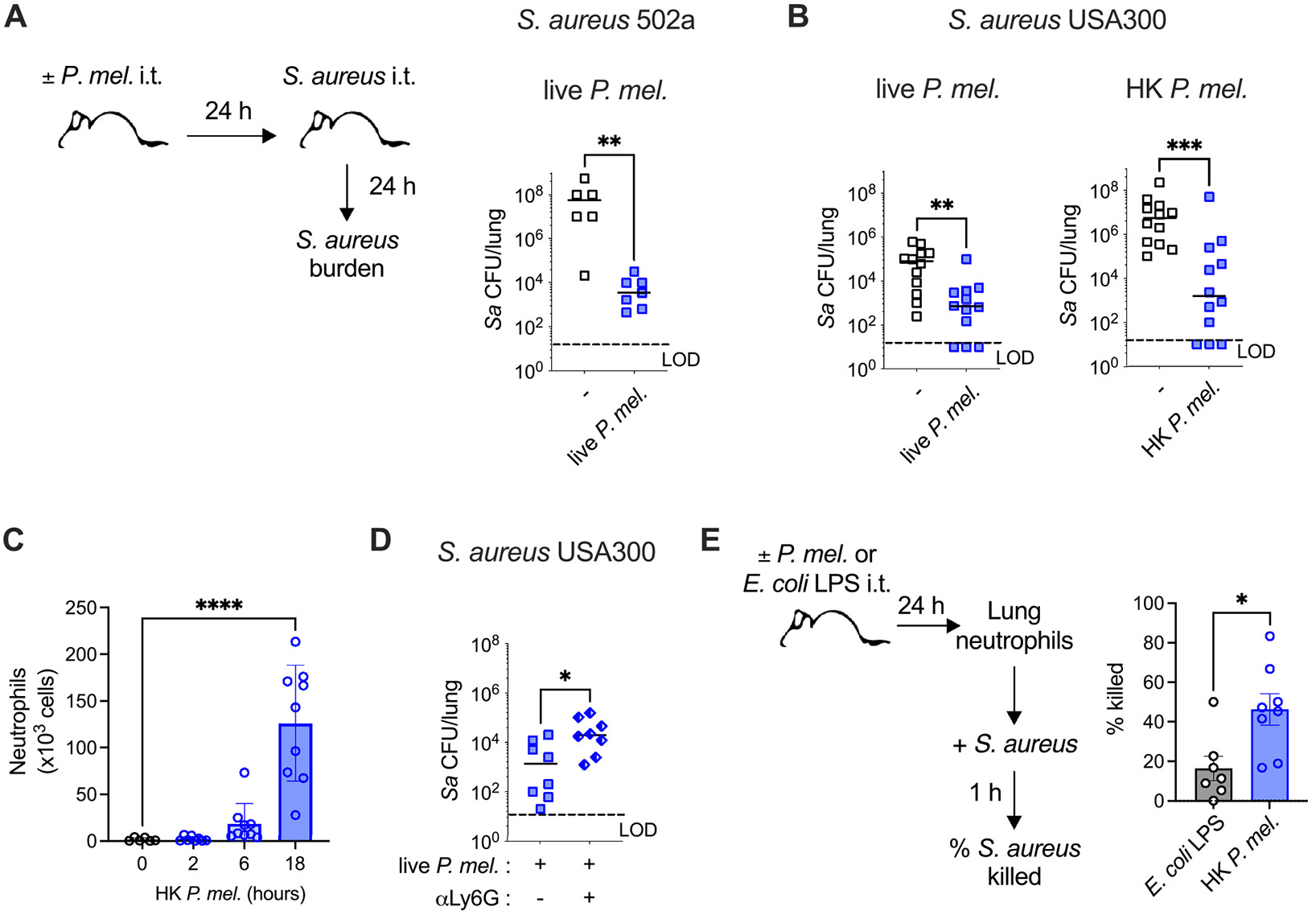
Exposure to *P. melaninogenica* reduces *S. aureus* lung infection and enhances neutrophil-mediated protection. A *S. aureus* lung burdens from BAL and lung tissue homogenates in wild-type mice with or without pre-exposure to *P. melaninogenica* (10^7^ CFU/mouse i.t.) 24 h prior to challenge with live *S. aureus* strain 502a (5 × 10^8^ CFU/mouse i.t.), (*n* = 6 mice/group). B *S. aureus* lung burdens in mice with or without pre-exposure to live (left) or heat-killed (right) *P. melaninogenica* (10^7^ CFU/mouse or CFU equivalents/mouse i.t.) 24 h prior to challenge with *S. aureus* strain USA300 (5 × 10^8^ CFU/mouse i.t.), (*n* = 12 mice/group). C Total numbers of neutrophils (Ly6G^+^CD11b^+^ cells) detected by flow cytometry in lung tissue in naïve mice and at the indicated time points post-exposure to heat-killed *P. melaninogenica* (*n* = 6–9 mice/group). D *S. aureus* lung burdens in mice pre-treated with anti-Ly6G antibody to deplete neutrophils, compared to mice treated with isotype control antibody (−), 24 h prior to live *P. melaninogenica* exposure followed by challenge with *S. aureus* strain USA300 as in (B), (*n* = 8 mice/group). E Percent *S. aureus* killed by neutrophils purified from mice treated with *E. coli* LPS or heat-killed *P. melaninogenica* for 24 h (*n* = 7 independent replicates/group). LOD indicates limit of detection. Data pooled from two (A) to three (B-E) independent experiments, displayed as mean ± SEM. *p<.05, **p<.01, ***p<.001, ****p<.0001, Mann-Whitney *U test* (A, B, D), unpaired *t-*test (E), one-way ANOVA (C).

To investigate the effect of *Prevotella* exposure on defense against *S. aureus* infection in a non-CF background, wild-type (WT) mice were pre-exposed to *P. melaninogenica* (intratracheally, i.t.) followed by *S. aureus* challenge (Fig. 1A). In mice pre-exposed to live *P. melaninogenica*, lung burdens of *S. aureus* were significantly reduced at 24 h post-infection, compared to those infected with *S. aureus* alone (Fig. 1A). This phenotype was consistent across two *S. aureus* strains; 502a and USA300, where *P. melaninogenica* pre-exposure reduced *S. aureus* lung burdens by several logs (Fig. 1B). Of note, mice were exposed to over a log lower dose of *P. melaninogenica* than *S. aureus*, in alignment with total anaerobic bacterial burdens reported as ~1 log lower than burdens of aerobic bacteria in sputum from pwCF [28]. While there is limited culture-based analysis of anerobic bacterial concentrations in the lungs of pwCF, in one study *P. melaninogenica* ranged from 10^5^ to 9 × 10^7^ colony forming units (CFUs) per gram of sputum, consistent with total anaerobic burdens of up to 10^8^ CFUs/gram sputum in another report [28,35]. A similar protective effect against *S. aureus* USA300 infection was observed following pre-exposure to heat-killed *P. melaninogenica* (Fig. 1B), suggesting an indirect mechanism. These findings suggest that in a non-CF environment, *P. melaninogenica* exposure improves early defense against acute *S. aureus* infection.

Given the critical role of neutrophils for bacterial defense, the impact of *P. melaninogenica* exposure on neutrophil recruitment and *S. aureus* clearance were evaluated. Heat-killed *P. melaninogenica* induced the recruitment of neutrophils to the lungs, with significantly increased total numbers by 18 h post-exposure (Fig. 1C). To determine whether the recruited neutrophils contribute to *P. melaninogenica*-mediated protection, mice were treated with anti-Ly6G antibody to deplete neutrophils prior to *P. melaninogenica* exposure and *S. aureus* infection (Supplemental Figure 1A). Burdens of *S. aureus* were significantly elevated in neutrophil-depleted mice despite *P. melaninogenica* pre-exposure, compared to lower *S. aureus* burdens in isotype control treated mice pre-exposed to *P. melaninogenica*, indicating an important role for neutrophils in *P. melaninogenica*-enhanced defense against *S. aureus* (Fig. 1D). To investigate the effect of *P. melaninogenica* pre-exposure on neutrophil function, neutrophil killing of *S. aureus* was assessed *ex vivo*. Neutrophils from the lungs of *P. melaninogenica* exposed mice were compared to those isolated from mice exposed to *Escherichia coli* lipopolysaccharide (LPS), which induces neutrophil recruitment to the lungs [20] but unlike *P. melaninogenica* was not protective against *S. aureus* infection (Supplemental Figure 1B). Neutrophils isolated from the lungs of *P. melaninogenica* exposed mice exhibited significantly enhanced *S. aureus* killing relative to neutrophils isolated from the lungs of LPS exposed controls (Fig. 1E), suggesting that *P. melaninogenica* exposure improved neutrophil-mediated clearance of *S. aureus*.

### 2.2. P. melaninogenica-induced cytokine production is higher in CFTR functional epithelial cells than in CFTR mutant epithelial cells

**Fig. 2.**
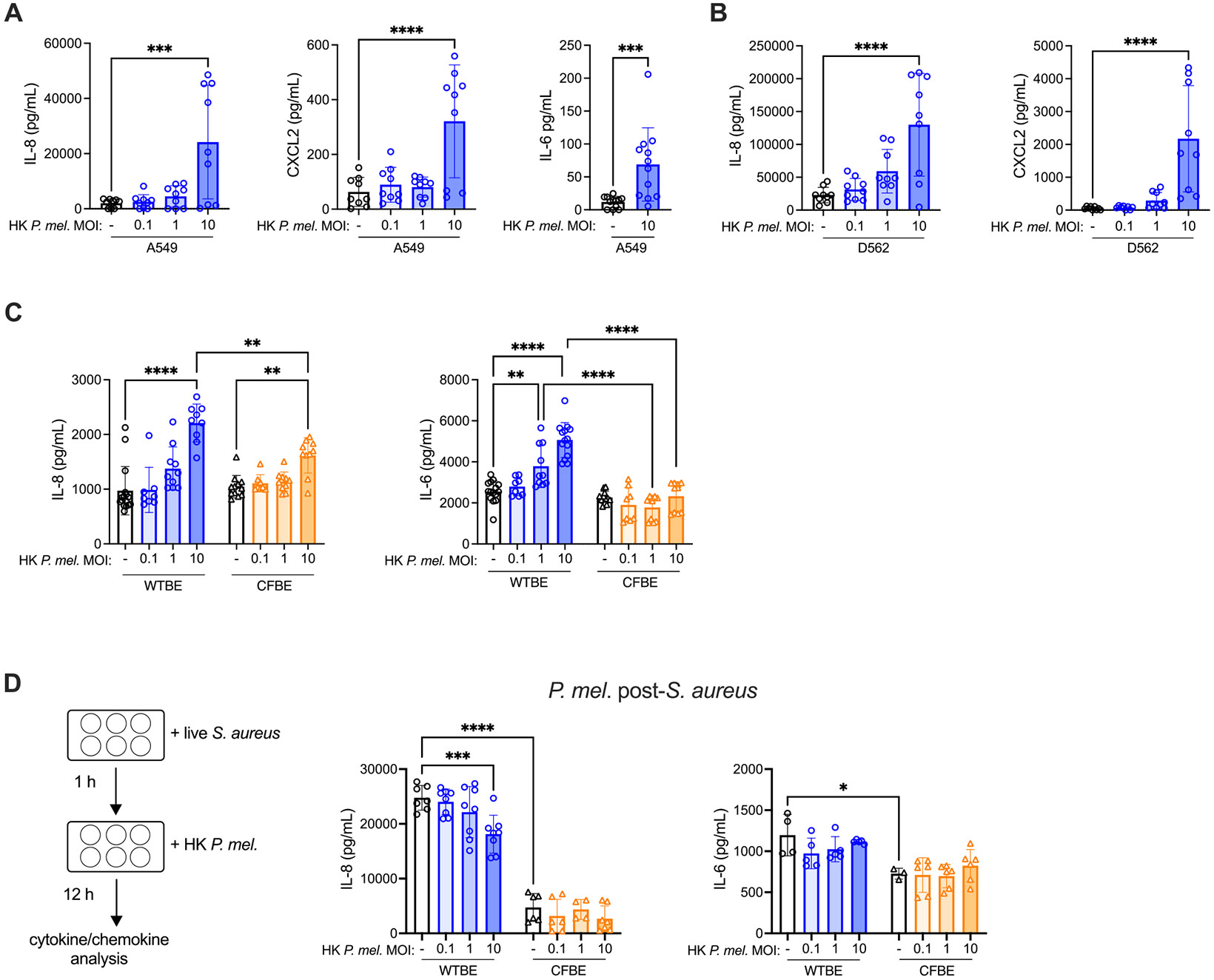
*P. melaninogenica* stimulation of cytokine and chemokine production by CFTR functional and deficient respiratory tract epithelial cells. A Supernatant IL-8, CXCL2, and IL-6 detected following 24-hour exposure to heat-killed *P. melaninogenica* at the indicated multiplicity of infection (MOI) in A549 alveolar epithelial cells. B Supernatant IL-8 and CXCL2 detected following 24-hour exposure to heat-killed *P. melaninogenica* at the indicated MOIs in D562 pharyngeal epithelial cells. C Supernatant IL-8 and IL-6 detected following 24-hour exposure to heat-killed *P. melaninogenica* in WT corrected (WTBE) cells and isogenic CFTR mutant (CFBE) cells. D Supernatant IL-8 and IL-6 detected in cells infected with live *S. aureus* for 1 h followed by the addition of heat-killed *P. melaninogenica* for 12 h co-incubation in WTBE cells and CFBE cells. Data pooled from three independent experiments displayed as mean ± SEM. **p<.01, ***p<.001, ****p<.0001, one-way ANOVA or Mann-Whitney U test (A-B), two-way ANOVA with Sidak’s *post-hoc* analysis (C-D).

We next investigated the impact of *P. melaninogenica* on respiratory tract epithelial cells, which are a critical first line of defense against pathogen acquisition and important regulators of neutrophil recruitment and function. Epithelial secretion of the neutrophil recruiting chemokines IL-8 and CXCL2 and the cytokine IL-6, which positively regulates neutrophil recruitment and function [36], were analyzed following 24 h *P. melaninogenica* exposure. In both A549 alveolar epithelial cells and D562 pharyngeal epithelial cells, heat-killed *P. melaninogenica* elicited a dose-dependent increase in the chemokines IL-8 and CXCL2, and significantly increased IL-6 production in A549 cells (Fig. 2A-B). To determine the relevance of CFTR function for these responses, IL-8 and IL-6 secretion in CFTR mutant bronchial epithelial cells (ΔF508/ΔF508 genotype CFBE41o- cells, CFBE) were compared to isogenic WT corrected CFBE41o- cells (WTBE), which stably express WT CFTR. While heat-killed *P. melaninogenica* induced a dose-dependent increase in IL-8 secretion in both cell lines, the IL-8 response was higher in WTBE cells compared to CFTR mutant (CFBE) cells (Fig. 2C). IL-6 production was also significantly higher in WTBE cells exposed to *P. melaninogenica* compared to CFTR mutant cells, in which *P. melaninogenica* exposure had a minimal effect on IL-6 (Fig. 2C).

To evaluate the effect of *P. melaninogenica* exposure on *S. aureus*-induced epithelial activation, heat-killed *P. melaninogenica* was introduced at 1-hour post-*S. aureus* infection for a 12-hour co-incubation. In WTBE cells, exposure to *P. melaninogenica* after *S. aureus* infection resulted in decreased IL-8 secretion, while IL-6 production was unchanged (Fig. 2D). IL-8 and IL-6 secretion were both lower in CFBE cells compared to WT corrected cells in response to *S. aureus* infection, with no further decrease during co-incubation (Fig. 2D). Together, these findings indicate that epithelial cytokine and chemokine responses to *Prevotella* are higher in CFTR functional cells.

### 2.3. P. melaninogenica reduces adherence of S. aureus to epithelial cells with functional CFTR

**Fig. 3.**
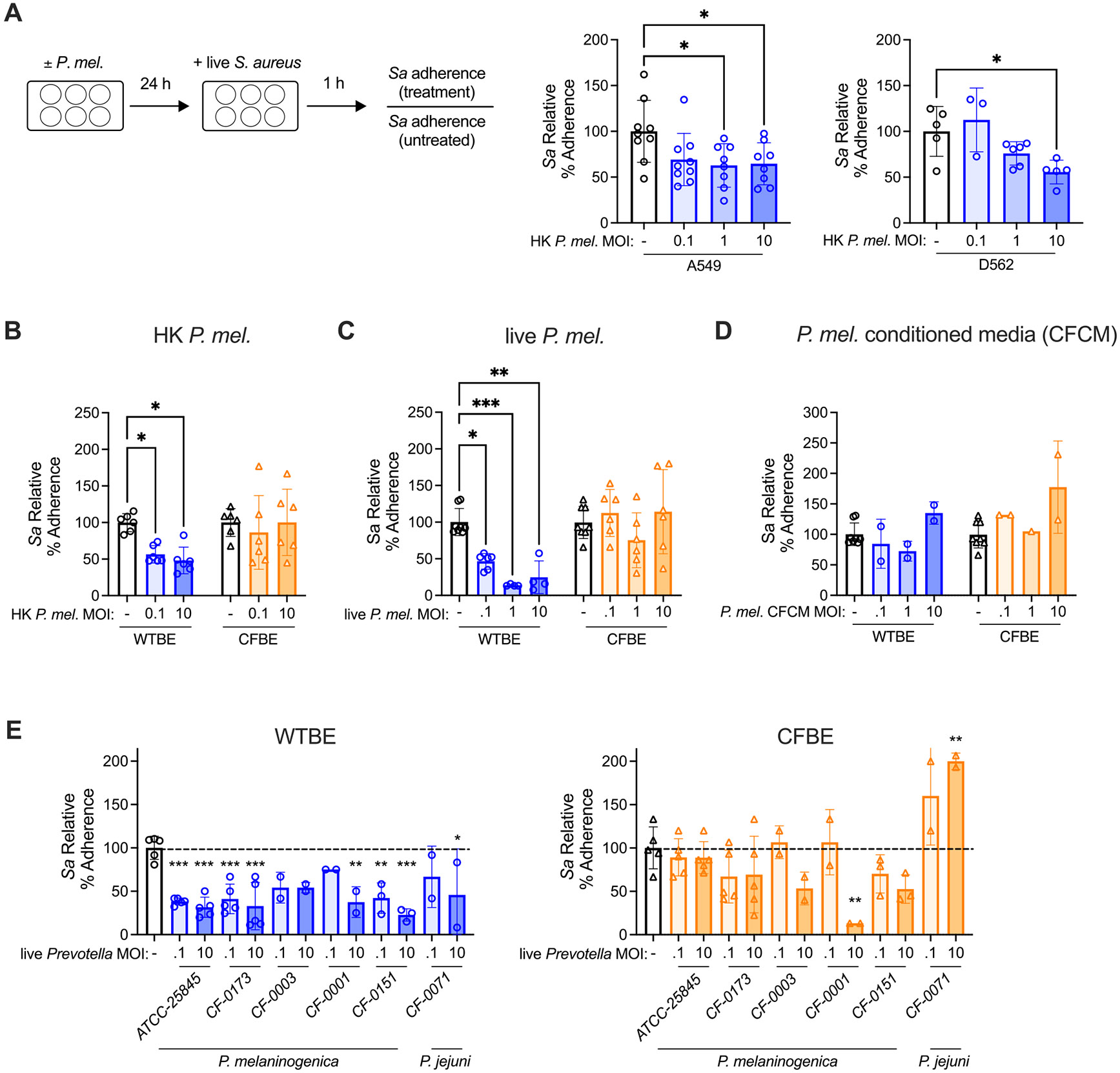
*P. melaninogenica* reduces *S. aureus* adherence to airway epithelial cells. A *S. aureus* relative adherence to A549 alveolar epithelial cells and D562 pharyngeal epithelial cells following 24-hour pre-exposure to heat-killed *P. melaninogenica*. B-D *S. aureus* relative adherence to WTBE and CFTR mutant (CFBE) cells following 24-hour pre-exposure to heat-killed *P. melaninogenica* (B), live *P. melaninogenica* (C), or cell free conditioned media from *P. melaninogenica* (D). E *S. aureus* relative adherence to WTBE and CFBE cells following 24-hour pre-exposure to live *P. melaninogenica* (ATCC 25,845) or clinical isolates of *P. melaninogenica* or *P. jejuni* from the sputum of pwCF. Data pooled from two (D, E) or three (A, B, C) independent experiments displayed as mean ± SEM. *p<.05, **p<.01, ***p<.001, ****p<.0001, one-way ANOVA with Dunnett’s *post-hoc* analysis.

The impact of *P. melaninogenica* exposure on *S. aureus* epithelial colonization was examined using adherence assays, as effective epithelial adherence is a critical early step for establishing *S. aureus* infection. Pre-exposure to heat-killed *P. melaninogenica* significantly reduced *S. aureus* adherence to both A549 and D562 respiratory tract epithelial cells in a dose-dependent manner, with a ~50 % reduction in *S. aureus* adherence at a multiplicity of infection (MOI) of 10 heat-killed *P. melaninogenica* (Fig. 3A). While baseline *S. aureus* adherence to WTBE and CFBE cells was similar (Supplemental Figure 2A), significant differences emerged following *P. melaninogenica* preexposure. In WTBE cells, pre-exposure to heat-killed *P. melaninogenica* significantly reduced *S. aureus* adherence (Fig. 3B). However, this protective effect was absent in CFTR mutant (CFBE) cells under identical conditions (Fig. 3B). Similarly, pre-exposure to live *P. melaninogenica* reduced *S. aureus* adherence to WTBE cells by over 75 %, but *S. aureus* adherence was unaffected by live *P. melaninogenica* exposure in CFBE cells (Fig. 3C). In contrast to heat-killed and live *Prevotella*, cell-free conditioned media (CFCM) from *P. melaninogenica* cultures had a minimal impact on *S. aureus* adherence (Fig. 3D).

To explore the range of *Prevotella* strains capable of reducing *S. aureus* adherence to respiratory tract epithelial cells, clinical isolates derived from the sputum of pwCF were surveyed, including four isolates of *P. melaninogenica* and one isolate of the closely related species *Prevotella jejuni*. Clinical *P. melaninogenica* isolates largely recapitulated findings with the original strain (ATCC 25,845), with three out of four *P. melaninogenica* isolates and the *P. jejuni* isolate capable of significantly reducing *S. aureus* adherence in WTBE cells. In contrast, all but one *P. melaninogenica* isolate had a limited effect on *S. aureus* adherence in CFTR mutant (CFBE) cells, and *S. aureus* adherence was significantly elevated following pre-exposure to the *P. jejuni* isolate (Fig. 3E). These findings indicate that *P. melaninogenica* impairs *S. aureus* adherence to CFTR functional epithelial cells.

### 2.4. TLR2 is required for P. melaninogenica-enhanced defense against S. aureus

**Fig. 4.**
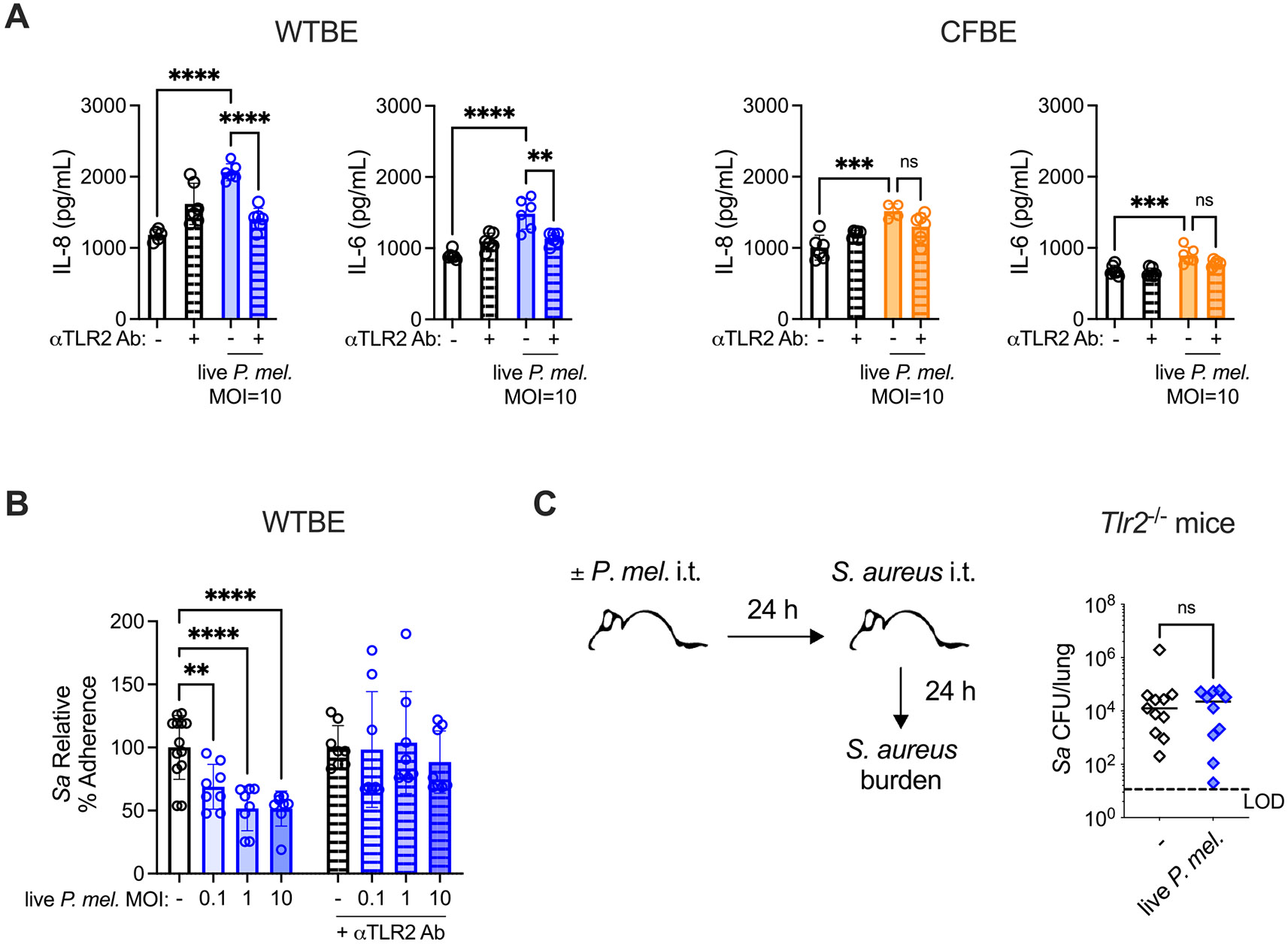
TLR2 is required for *P. melaninogenica*-induced impairment of *S. aureus* adherence and lung infection. A Supernatant IL-8 and IL-6 detected 24 h following exposure to live *P. melaninogenica* in WTBE cells and CFTR mutant (CFBE) cells treated with anti-TLR2 antibody to block TLR2 signaling, compared to cells treated with isotype control antibody. B *S. aureus* relative adherence to WTBE cells following 24-hour pre-exposure to live *P. melaninogenica* with or without anti-TLR2 antibody. C *S. aureus* lung burdens in *Tlr2*^−/−^ mice at 24 h post-infection with or without pre-exposure to live P. *melaninogenica* (*n* = 10–11 mice/group). LOD indicates limit of detection. Pooled from two (C) or three (A-B) independent experiments displayed as mean ± SEM. **p<.01, ***p<.001, ****p<.0001, one-way ANOVA with Dunnett’s *post-hoc* analysis (A-B), Mann-Whitney U test (C).

As *Prevotella* have been shown to activate epithelial TLR2 [37] and TLR2 was required for *P. melaninogenica*-enhanced defense against *S. pneumoniae* infection [20], we next evaluated the importance of TLR2 for *P. melaninogenica*-mediated effects on *S. aureus*. Epithelial cells were treated with a TLR2 antagonist antibody (clone TL2.1), which reduced IL-8 and IL-6 production by WTBE cells stimulated with the TLR2 agonist Pam3SK4 (Supplemental Figure 2B). IL-8 and IL-6 production in response to live *P. melaninogenica* were reduced to baseline levels in WTBE cells treated with anti-TLR2 antibody compared to cells treated with isotype control antibody (Fig. 4A). Consistent with the limited response to *P. melaninogenica* in CFTR mutant (CFBE) cells, anti-TLR2 antibody treatment had a minimal effect on IL-8 and IL-6 secretion in CFBE cells (Fig. 4A).

To determine the importance of TLR2 for *P. melaninogenica*-mediated impairment of *S. aureus* adherence, epithelial cells were treated with anti-TLR2 antibody or isotype control antibody during *P. melaninogenica* pre-exposure and *S. aureus* infection. Anti-TLR2 antibody and isotype control antibody had no impact on *S. aureus* adherence in the absence of *P. melaninogenica* pre-exposure (Supplemental Figure 2C). However, anti-TLR2 antibody treatment completely abrogated the protective effect of live *P. melaninogenica* pre-exposure against *S. aureus* adherence (Fig. 4B), in contrast to both untreated cells and cells treated with isotype control antibody, where *P. melaninogenica* impairment of *S. aureus* adherence was maintained (Fig. 4B, Supplemental Figure 2D). Loss of *P. melaninogenica*-mediated impairment of *S. aureus* adherence was confirmed in a second anti-TLR2 antibody antagonist (clone 383,936) (Supplemental Figure 2E-F). To determine the importance of TLR2 for *P. melaninogenica*-induced defense against *S. aureus* infection in vivo, the impact of *P. melaninogenica* exposure on *S. aureus* lung infection was evaluated in TLR2-deficient mice. In contrast to WT mice, *S. aureus* burdens in TLR2-deficient mice were similar regardless of *P. melaninogenica* pre-exposure (Fig. 4C). Together, these data highlight an important role for TLR2 mediating *P. melaninogenica*-induced protection against *S. aureus* adherence and infection.

### 2.5. Short-chain fatty acids modulate the epithelial cytokine response to P. melaninogenica

*Prevotella* species produce short-chain fatty acids (SCFAs) including butyrate and propionate as part of anaerobic metabolism [38]. The effect of exogenous SCFAs on epithelial responses was evaluated as a surrogate for elevated *Prevotella*-mediated SCFA production in high mucus environments such as the CF lungs. In WTBE cells, the SCFA butyrate increased baseline IL-8 secretion and propionate significantly elevated IL-8 levels following exposure to heat-killed *P. melaninogenica* (Supplemental Figure 3A). SCFA effects were similar in CFTR mutant (CFBE) cells, where butyrate increased IL-8 secretion in response to *P. melaninogenica* (Supplemental Figure 3A). In contrast, IL-6 secretion in WTBE and CFBE cells was significantly reduced by butyrate and propionate exposure (Supplemental Figure 3B), both at baseline and following *P. melaninogenica* exposure. Similar trends were observed for IL-8 and IL-6 secretion in response to the TLR2 agonist Pam3SK4, as butyrate and propionate increased Pam3SK4-induced IL-8 secretion but suppressed the IL-6 response to Pam3SK4 in WTBE and CFBE cells (Supplemental Figure 3C-D).

In contrast to the modulatory effects of SCFAs on *P. melaninogenica*-induced IL-8 and IL-6 production, neither butyrate nor propionate had a significant effect on *P. melaninogenica*-mediated impairment of *S. aureus* adherence (Supplemental Figure 3F). In WTBE cells, pre-exposure to heat-killed *P. melaninogenica* significantly reduced *S. aureus* adherence regardless of SCFA co-exposure, despite a trend toward elevated baseline *S. aureus* adherence in cells treated with propionate (Supplemental Figure 3F). The minimal effect of *P. melaninogenica* exposure on *S. aureus* adherence in CFTR mutant (CFBE) cells was maintained in butyrate or propionate exposed cultures, though there was a slight (non-significant) reduction in adherence for cells treated with propionate and exposed to *P. melaninogenica* (Supplemental Figure 3F). Together, these data indicate a modulatory effect for SCFAs on *P. melaninogenica*-induced cytokine responses but not *P. melaninogenica*-mediated impairment of *S. aureus* adherence in CFTR functional cells.

### 2.6. P. melaninogenica selectively upregulates antibacterial defense pathways in CFTR functional epithelial cells

Due to the observed differences in *P. melaninogenica*-mediated effects on cytokine production and pathogen adherence between WTBE and CFTR mutant (CFBE) cells, RNA sequencing was performed on untreated cells and on cells following *P. melaninogenica* exposure. Consistent with a recent study comparing the transcriptional profiles of WTBE and CFBE cells at baseline [39], there were large transcriptional differences between untreated WTBE and CFBE cells, with enrichment in gene ontology terms related to antimicrobial defense including cytokine-mediated signaling, response to bacterium, and defense response to virus in WT corrected cells (Supplemental Figure 4A). While functional defects due to CFTR mutation are caused by CFTR protein misfolding, *CFTR* expression in CFBE cells was also low compared to WTBE cells (Supplemental Figure 4B), as previously reported [39,40].

**Fig. 5.**
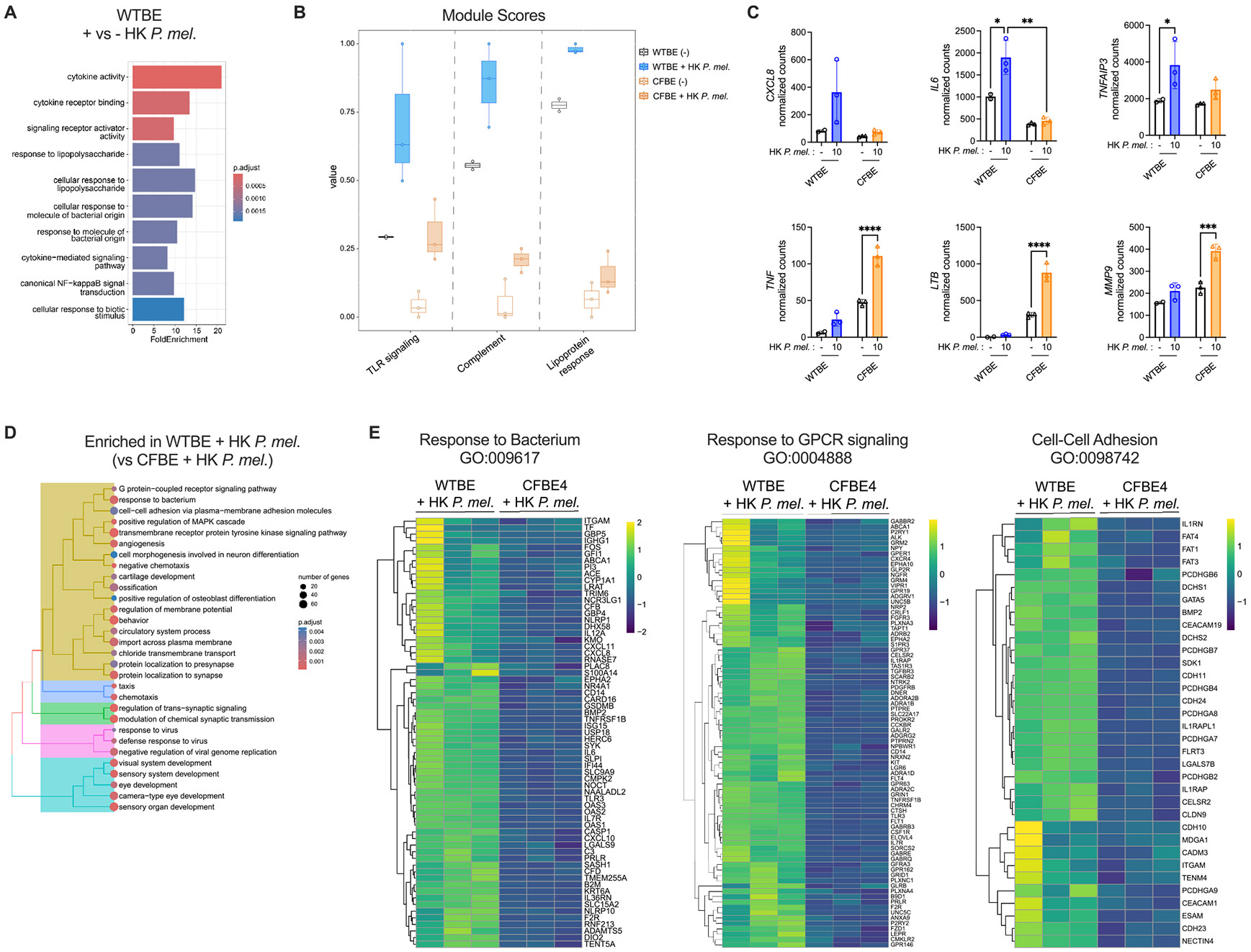
*P. melaninogenica*-induced antimicrobial response and immune defense signaling pathways in epithelial cells are CFTR dependent. A Enrichment of the top ten significant differentially expressed gene ontology (GO) terms in WTBE cells exposed to heat-killed *P. melaninogenica* for 24 h, compared to untreated WTBE cells. B Module scoring for TLR signaling, complement, and lipoprotein response associated genes in WTBE cells and CFTR mutant (CFBE) cells with or without exposure to heat-killed *P. melaninogenica*. C Normalized gene counts for *CXCL8* (IL-8), *IL6, TNFAIP3, TNF, LTB*, and *MMP9* in WTBE cells and CFBE cells with or without exposure to heat-killed *P. melaninogenica.* D Tree plot of top 30 significant GO terms in WTBE cells exposed to *P. melaninogenica* compared to CFBE cells exposed to *P. melaninogenica*. E Heat maps of all significant differentially expressed genes from the indicated GO terms for WTBE versus CFBE cells exposed to *P. melaninogenica*. Gene count data displayed as individual points plus mean ± SEM. *p<.05, ***p<.001, ****p<.0001, one-way ANOVA with Sidak’s *post-hoc* analysis.

In WTBE cells exposed to *P. melaninogenica*, the top ten significant gene ontology terms all related to cytokine activity, response to lipids and molecules of bacterial origin, and canonical NFκB signaling (Fig. 5A). While *P. melaninogenica* also altered the transcriptional profile of CFTR mutant (CFBE) cells, the pattern of gene expression differed from WT corrected cells, with distinct top upregulated and down-regulated genes (Supplemental Figure 4C-D, Supplemental Table 1). To explore patterns of custom gene set expression, we employed a module scoring function to assess expression of the gene set in aggregate on a per sample basis. Module scoring for genes related to TLR signaling, complement, and lipoprotein response revealed a pattern of higher baseline expression in WTBE cells and overall greater induction following *P. melaninogenica* exposure compared to CFTR mutant (CFBE) cells (Fig. 5B). Consistent with elevated IL-8 and IL-6 protein secretion induced by *P. melaninogenica* in WTBE cells, gene expression of *CXCL8* (encoding IL-8) and *IL6* was also higher in WT corrected cells compared to CFBE cells treated with *P. melaninogenica* (Fig. 5C). A similar pattern was observed for *TNFAIP3*, an important regulator of NFκB (Fig. 5C). However, not all immune and microbial response genes followed this pattern, as CFBE cells had significantly higher expression of *TNF* (a pro-inflammatory cytokine), *LTB* (TNF family), and *MMP9* (degrades extracellular matrix proteins) following *P. melaninogenica* exposure compared to WT corrected cells (Fig. 5C).

Based on the observation that TLR2 signaling is required for *P. melaninogenica*-induced effects in CFTR functional epithelial cells, the expression of *TLR2* and the co-receptor for lipoprotein recognition, *TLR1*, were also compared. *TLR2* expression was largely unaltered regardless of *P. melaninogenica* exposure or CFTR function (Supplemental Figure 4E). *TLR1* was low at baseline in CFTR mutant (CFBE) cells compared to WT corrected cells but increased to a similar level in CFBE cells in *P. melaninogenica* exposed samples (Supplemental Figure 4E). Of note, CFBE41o- cells carry a single nucleotide polymorphism (SNP Ile602ser) in *TLR1* which reduces protein function [41], though some function is retained. This mutation is extremely common in pwCF, and 60–70 % of all American and Canadian Caucasians are homozygous for this SNP [41,42].

Gene ontology (GO) analysis of transcriptional differences between WTBE and CFBE cells exposed to *P. melaninogenica* revealed several pathways which were significantly enriched in WT corrected cells related to microbial defense including G protein-coupled receptor (GPCR) signaling, response to bacterium, regulation of mitogen-activated protein kinase (MAPK) pathway, and cell-cell adhesion (Fig. 5D, Supplemental Table 2). In contrast, overrepresented pathways in CFBE cells exposed to *P. melaninogenica* were limited to cytosolic small ribosomal subunit associated genes (Supplemental Figure 4F). Differential expression analysis confirmed broad upregulation among genes from GO terms for response to bacterium, response to GPCR signaling, and cell-cell adhesion in WTBE cells exposed to *P. melaninogenica*, compared to overall lower expression in CFBE cells exposed to *P. melaninogenica* (Fig. 5E). Similar patterns were observed for genes from the GO term for regulation of MAPK signaling (Supplemental Figure 4G), which occurs downstream of TLR2 activation. Fibronectin (*FN1*) expression was also compared, which binds *S. aureus* adhesin proteins [43]. *FN1* was expressed at high levels, regardless of CFTR function or *Prevotella* exposure (Supplemental Figure 4H), indicating a minimal contribution for fibronectin transcriptional regulation. GPCRs encompass a large family of surface receptors, several which have important roles in CFTR regulation [44-47]. Among these, the A2B adenosine receptor (*A2B*) and the β2 adrenergic receptor (*ADRB2*) are well-established CFTR regulators and highly expressed in the airways [48,49]. Both *A2B* and *ADRB*2 were significantly elevated in WTBE cells compared to CFBE cells, though *Prevotella* exposure didn’t alter expression in either cell type (Supplemental Figure 4I). Genes related to ion transport, which is directly tied to CFTR function, and genes from the GO term for response to virus were also broadly elevated in WTBE cells exposed to *P. melaninogenica* compared to CFBE cells (Supplemental Figure 4J-K). A similar pattern was observed for genes related to wound repair, which were generally elevated in WTBE but not CFBE cells exposed to *P. melaninogenica*, while expression of genes involved in apoptosis signaling was mixed, with a different set of genes elevated in WTBE versus CFBE cells exposed to *P. melaninogenica* (Supplemental Figure 4K). Together, these data indicate large differences in the transcriptional response to *P. melaninogenica* tied to CFTR function, with greater antimicrobial defense induction in cells with functional CFTR expression.

### 2.7. CFTR modulator therapy rescues P. melaninogenica-induced defense against S. aureus adherence in CFTR mutant cells

**Fig. 6.**
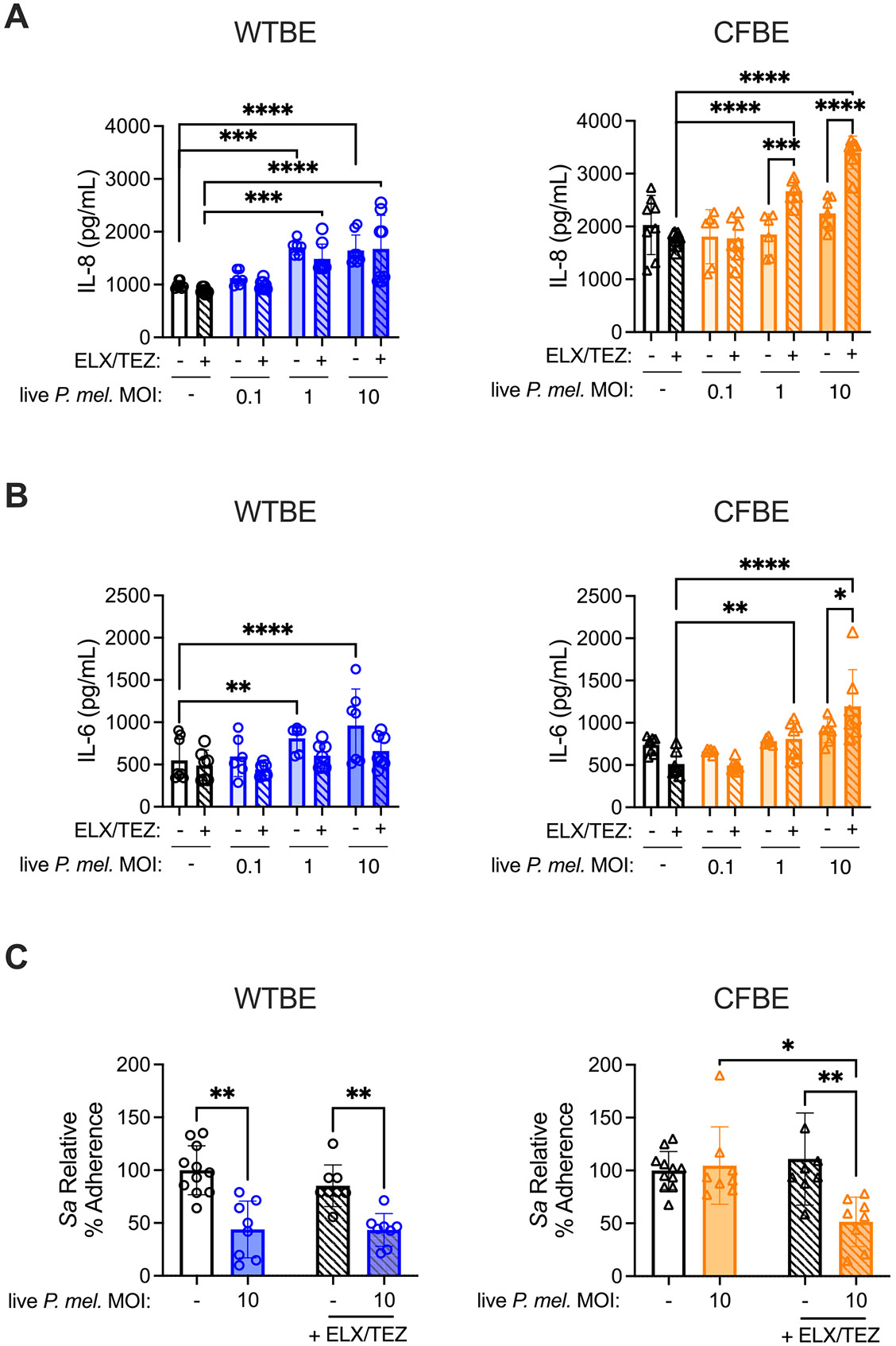
CFTR modulators increase *P. melaninogenica*-induced cytokine production and impairment of *S. aureus* adherence in CFTR mutant cells. A-B Supernatant IL-8 (A) and IL-6 (B) detected in WTBE and CFTR mutant (CFBE) cells exposed to live *P. melaninogenica* in the presence or absence of the CFTR modulators elexacaftor (0.3 μM) and tezacaftor (0.2 μM) for 24 h. C *S. aureus* relative adherence to WTBE and CFBE cells following 24-hour pre-exposure to live *P. melaninogenica* in the presence or absence of elexacaftor and tezacaftor for 24 h. Data pooled from three independent experiments displayed as mean ± SEM. *p<.05, **p<.01, ***p<.001, ****p<.0001, two-way ANOVA with Sidak’s *post-hoc* analysis (A-B), one-way ANOVA with Dunnett’s *post-hoc* analysis (C).

To better understand how CFTR influences the epithelial response to *P. melaninogenica*, and if *P. melaninogenica*-mediated protective effects could be restored with functional CFTR, CFBE epithelial cells were treated with CFTR modulators. Cells were treated with elexacaftor and tezacaftor, as ivacaftor reduces the rescue efficiency of elexacaftor/tezacaftor in CFBE41o- cells [50]. In WTBE cells, IL-8 and IL-6 secretion increased following exposure to live *P. melaninogenica*, independent of modulator therapy (Fig. 6A-B). In CFTR mutant (CFBE) cells, the subdued response to *P. melaninogenica* compared to WT corrected cells was reversed with CFTR modulator therapy, which significantly increased *P. melaninogenica*-induced IL-8 and IL-6 production in CFBE cells (Fig. 6A-B). Cytokine levels in cells not exposed to *P. melaninogenica* were unchanged by CFTR modulators.

The effect of CFTR modulator therapy on *P. melaninogenica*-mediated impairment of *S. aureus* adherence was also addressed in CFBE cells. In WT corrected cells, pre-exposure to live *P. melaninogenica* significantly reduced *S. aureus* adherence, and this effect was maintained in cells treated with CFTR modulators (Fig. 6C). In contrast, CFTR modulator therapy had a significant effect in CFTR mutant (CFBE) cells, rescuing *P. melaninogenica*-mediated impairment of *S. aureus* adherence, which was only observed in CFBE cells treated with live *P. melaninogenica* in the presence of CFTR modulators (Fig. 6C). Together, these data indicate that CFTR modulator therapy is sufficient to restore *P. melaninogenica*-mediated protection against *S. aureus* adherence in CFTR mutant epithelial cells.

## 3. Discussion

Despite significant advances in CF management with HEMT, persistent airway infection and inflammation remain major clinical concerns. Here, we uncovered a protective role for *P. melaninogenica* against *S. aureus* infection, demonstrating a mechanism by which oral commensal anaerobes may contribute to pathogen defense in pwCF on CFTR modulator therapy. *Prevotella* increase in abundance following HEMT [5] and anaerobe abundance correlates with improved lung function in pwCF on CFTR modulator therapy [4], highlighting the opportunity for *P. melaninogenica*-mediated effects in pwCF on CFTR modulators. A recent study of pwCF on HEMT demonstrated a sustained increase in sputum IL-6 while other inflammatory markers were reduced, highlighting the potential importance of IL-6 in pulmonary homeostasis for pwCF on HEMT [51]. Here, we demonstrate the potential for *P. melaninogenica* to contribute the regulation of IL-6 and epithelial antibacterial defense, which we show improve upon CFTR modulator treatment, in pwCF.

In people without CF, *Prevotella* abundance is linked with subclinical inflammation in the lower airway, including increased neutrophil activation [52,53]. Neutrophils are the first immune cells recruited to the lungs following infection or injury and serve as key players in host defense through activities including bacterial killing. Our data suggest that *P. melaninogenica* enhance this beneficial axis of neutrophil-mediated immunity in the context of *S. aureus* infection, consistent with prior findings demonstrating *P. melaninogenica*-enhanced protection against *S. pneumoniae* [20]. These effects appear to be species-specific, as in contrast to *P. melaninogenica, Prevotella intermedia* was reported to reduce neutrophil-mediated killing and exacerbate *S. aureus* infection in mice [54]. In the lungs of pwCF, bacterial clearance remains inefficient despite overwhelming neutrophilic infiltration, with neutrophil dysfunction linked to the suppression of reactive oxygen species (ROS) and phagocytic killing due to CFTR deficiency and extracellular mucins [55,56]. Neutrophil-intrinsic expression of CFTR, not addressed in the current study, may have additional consequences regarding direct and indirect *P. melaninogenica*-induced activation. While our data indicate a beneficial impact for *Prevotella*-mediated neutrophil recruitment and activation on bacterial clearance in the absence of CFTR mutation, it remains unclear whether HEMT is sufficient to restore neutrophil antimicrobial function and *P. melaninogenica*-enhanced activation to support pathogen clearance in the lungs of pwCF.

Regardless of the potential for *Prevotella*-enhanced neutrophil function in pwCF, our findings indicate a second mechanism by which *P. melaninogenica* exposure may improve defense against *S. aureus* through interactions with the respiratory tract epithelium. Unexpectedly, we found that *P. melaninogenica* exposure reduced *S. aureus* adherence to CFTR functional epithelial cells, including WTBE cells and CFBE cells treated with CFTR modulators. While cell-free supernatants from *P. melaninogenica* had no impact on *S. aureus* adherence, a prior report demonstrated that *Prevotella* supernatants were sufficient to induce IL-6 and IL-8 production in CFBE41o- epithelial cells [30], suggesting that distinct factors contribute to impaired *S. aureus* adherence. The limited effect of SCFAs on *P. melaninogenica* impairment of *S. aureus* adherence provides further evidence for divergence between the requirements for *P. melaninogenica*-induced cytokine secretion and reduced *S. aureus* adherence, beyond a conserved requirement for TLR2. *P. melaninogenica* exposure induced expression of a large family of cell-cell adhesion genes in WT corrected but not CFTR mutant (CFBE) cells, which may influence *S. aureus* adherence. *S. aureus* relies on fibronectin binding proteins to adhere to respiratory tract epithelial cells [57,58], and while fibronectin gene expression was unaffected by *P. melaninogenica* exposure, it isn’t clear whether *P. melaninogenica* modifies *S. aureus* fibronectin binding through other mechanisms. SCFAs were recently shown to disrupt *S. aureus* membrane integrity [59], indicating a parallel mechanism by which *P. melaninogenica*-derived products may interfere with *S. aureus* colonization. Several studies have characterized the direct interactions between *Prevotella* and CF pathogens, demonstrating that *Prevotella* cell-free supernatants interfere with *P. aeruginosa* biofilm formation [60] while live *Prevotella* contribute to *P. aeruginosa* cross-feeding [61,62]. *Prevotella* also alter pathogen-induced activation of epithelial cells, as *P. melaninogenica* reduced *P. aeruginosa*-induced IL-8 secretion in a lung epithelial organoid model [63] and other *Prevotella* species reduced *P. aeruginosa*-induced cytokine responses in CFBE41o- cells [30], consistent with our observation that *P. melaninogenica* reduced *S. aureus*-induced IL-8. These findings highlight the capacity for *Prevotella* components to alter epithelial responses, directly and indirectly influencing CF pathogen defense.

RNA sequencing revealed significant differences in the transcriptional response to *P. melaninogenica* in CFTR mutant versus WT corrected cells. Compared to WT corrected cells, CFBE cells had reduced baseline expression and lower *P. melaninogenica*-induced responses in several important antibacterial defense pathways including TLR signaling and lipoprotein responses, consistent with the TLR2 requirement for *P. melaninogenica*-mediated effects in CFTR functional cells. In addition to TLR2 activation by lipoproteins, other *Prevotella* components may contribute to epithelial activation, as *Prevotella* activate TLR5 in epithelial cells [31] and a glycoprotein fraction of *Prevotella*, likely including the TLR5 agonist flagellin, induced IL-8 production by gingival keratinocytes [64]. While select genes were upregulated by *P. melaninogenica* exposure to a greater extent in CFBE cells compared to WT corrected cells, overall expression patterns suggested a broad defect in antibacterial responses in CFTR mutant cells, including reduced MAPK signaling, which is downstream of TLR2, which may contribute to reduced pathogen defense and loss of the putative benefits of epithelial priming by *P. melaninogenica*. Reduced epithelial defense against pathogens could ultimately exacerbate inflammation as bacterial numbers expand, virulence factors dysregulate immune cell activity, and epithelial-immune cell crosstalk is disrupted, contributing to the hyperinflammatory environment in the CF airway. CFTR dysfunction creates higher baseline intracellular stress, including increased oxidative stress and abnormal calcium signaling, impairing responses to external stimuli [65,66]. The restoration of *P. melaninogenica*-induced impairment of *S. aureus* adherence in CFBE cells treated with CFTR modulators underlines a critical role for CFTR function upstream of *P. melaninogenica*-mediated changes in epithelial cells which enhance antibacterial defense.

Limitations to these studies include the use of immortalized epithelial cell lines, which allowed for direct comparisons between isogenic WT and CFTR mutant cells but are not fully representative of CF epithelium. While WT corrected CFBE41o- cells overexpress CFTR, the *P. melaninogenica*-induced cytokine response and impact on *S. aureus* adherence in CFBE cells treated with CFTR modulator were similar to those in WT corrected cells, suggesting that CFTR overexpression didn’t have a major effect on these phenotypes. In vitro systems also fail to recapitulate the in vivo CF airway environment in terms of complex physiology, mucus and ion trafficking, and other microbial interactions. Future studies using primary cell air liquid interface (ALI) cultures and more complex organoid models are important next steps in understanding how *Prevotella* regulate epithelial cell activation. ALI cultures of CFBE41o- cells were previously reported to have reduced cytokine responses to *P. aeruginosa* compared to WT corrected cells [67], consistent with our findings in submerged CFBE cells. It should be noted that other CFTR functional cell lines including 16HBE14o- cells are less responsive to bacteria than CFBE41o- cells, including lower *P. melaninogenica*-induced IL-8 secretion [38], though these aren’t isogenic cell lines. Infection studies were limited to a CFTR functional mouse model, as improvements to CF lung infection models in mice remain in progress. We used an infection model that focuses on the early dynamics occurring during acute infection with *S. aureus,* which may differ from those contributing to chronic *S. aureus* infection and persistence in the lungs of pwCF, an important area for further study. While this study incorporated clinical *P. melaninogenica* strains from the lungs of pwCF, future work should investigate interactions with *S. aureus* isolates from pwCF, which may have different gene expression and virulence properties impacting *Staphylococcal*-host interactions. Model systems capturing greater microbial complexity will also be important to understand the synergistic and antagonistic impact of other microbial species on *Prevotella*-mediated effects on epithelial activation and *S. aureus* infection.

Here, we define a mechanism by which the prevalent commensal *P. melaninogenica* enhances host defense against *S. aureus* in a TLR2 dependent manner which requires functional CFTR, indicating *P. melaninogenica* as an immune modulatory bacterium capable of altering pathogen infection dynamics. Clinically, these findings suggest that CFTR modulators may help to restore the epithelial response to ‘beneficial’ commensal bacteria, which we propose contribute to the regulation of epithelial antibacterial defense.

## 4. Materials and methods

### 4.1. Animals

C57BL/6J (WT) and B6.129Tlr2^tm1Kir^ (*Tlr2*^−/−^ , C57BL/6 J background) mice were purchased from The Jackson Laboratory (stocks #000664, #004650). Mice were housed in standard laboratory conditions (light cycle of 14:10 light:dark hours, 72 ± 2°F, and 40 ± 10 % humidity) at the University of Colorado Office of Laboratory Animal Resources. Adult male and female mice aged 6–12 weeks were used for these studies.

### 4.2. Bacteria

*S. aureus* strains included the methicillin-resistant *S. aureus* (MRSA) isolate USA300 (AH1263) [68] and 502a [69]. *S. aureus* strains were grown from glycerol stocks into liquid Brain Heart Infusion (BHI) media (BD Difco, ThermoFisher Scientific, Waltham, MA) incubated at 37 °C with shaking at 200 rpm to mid-log phase under aerobic conditions. Bacteria were pelleted (10 min, ≥20,000 x g) and resuspended in phosphate buffered saline (PBS) for animal infections or Ham’s F-12 K (Kaighn’s) medium (ThermoFisher Scientific) for in vitro cell infection.

*P. melaninogenica* strain ATCC^®^ 25,845^™^ was obtained from the American Type Culture Collection (Manassas, VA). Clinical *P. melaninogenica* (CF-01,773, CF-0003, CF-0001, CF0071) and *Prevotella jejuni* (CF-0151) isolates were isolated from the sputum of pwCF, with species determined by 16S rRNA gene sequencing. *P. melaninogenica* was grown from glycerol stocks onto Brucella Agar plates containing 5 % sheep blood, hemin and vitamin K (ThermoFisher Scientific) at 37 °C for 72 h under anaerobic conditions. *P. melaninogenica* cultures were harvested from plates by adding PBS and gently scraping bacterial growth from the plate surface. Heat killed *P. melaninogenica* were prepared by incubating plate extracts resuspended in PBS at 65 ° C for 30 min. CFU equivalents per mL were determined by serial dilution of plate extracts prior to heat killing. *P. melaninogenica* supernatants were prepared from PBS plate growth extractions by filtering supernatants after pelleting (10 min, ≥20,000 x g) through a 0.22 μM filter.

### 4.3. Animal infections

Mice were infected with *P. melaninogenica* intratracheally (i.t.) with a single dose of 10^7^ CFU per mouse under inhaled isoflurane anesthesia. 24 h after exposure to *P. melaninogenica* or PBS (vehicle control), mice were infected with *S. aureus* i.t. with a single dose of 5 × 10^8^ CFU per mouse under inhaled isoflurane anesthesia. For neutrophil depletion, mice were injected with 200 μg/mouse anti-mouse Ly6G antibody (Bio X Cell, Lebanon, NH, clone 1A8, catalog #BE0017–1) or anti-mouse IgG2A isotype control antibody (Bio X Cell, clone C1.18, catalog #BE0085) intraperitoneally (i.p.) 24 h prior to *P. melaninogenica* or PBS exposures. Neutrophil depletion was confirmed by flow cytometry (see below). Following sacrifice at the indicated time points, bronchoalveolar lavage (BAL) fluid was collected through cannulated tracheas in 1 mL 1x PBS. Lung tissue was homogenized in 500 μL of 1x PBS using a Bullet Blender tissue homogenizer (Stellar Scientific, Baltimore, MD). Homogenates were centrifuged at 500 x g for 30 s to pellet tissue debris. BAL and lung homogenates were serially diluted and plated on selective agar for CFU quantification.

### 4.4. Flow cytometry

For flow cytometry, lung tissue was harvested following perfusion by transcardial injection of 10 mL PBS. Tissue was homogenized using mechanical digestion (mincing) and enzymatic digestion (DNAseI 30 ug/mL, Sigma-Aldrich, St. Louis, MO, and type 4 collagenase 1 mg/mL, Worthington Biochemical Corporation, Lakewood, NJ). Red blood cell (RBC) lysis buffer was used to lyse red blood cells (0.15 M NH_4_Cl, 10 mM KHC0_3_, 0.1 mM Na2EDTA, pH 7.4). Fc receptors were blocked using anti-mouse CD16/32 followed by live/dead staining (LIVE/DEAD^™^ Fixable cell stain kit, Invitrogen, ThermoFisher Scientific) prior to staining with anti-mouse Ly6G (BioLegend, San Diego, CA, catalog #127,614, clone 1A8), anti-mouse CD45.2 (BD Biosciences, Franklin Lakes, NJ, catalog #564,616, clone 104), anti-mouse CD11b (Bio-Legend, catalog #101,212, clone M1/70), and anti-mouse SiglecF (BD, catalog #562,681, clone E50–2440). Neutrophils were defined as CD45.2^+^, SiglecF^−^, Ly6G^+^CD11b^+^ cells as previously described [20]. Flow cytometry samples were analyzed on an LSR Fortessa X-20 in University of Colorado Flow Cytometry Shared Resource core (RRID: SCR_022035). Data analysis was performed with FlowJo software version 9.9.6 (BD Biosciences).

### 4.5. Neutrophil killing assay

Lung neutrophils were purified by positive selection using antibody labeled magnetic beads (anti-Ly6G-PE with MojoSort PE-positive selection kit, BioLegend), with purity of *>*90 % Ly6G^+^CD11b^+^ neutrophils confirmed by flow cytometry. Neutrophils were isolated from lung tissue 24 h post-exposure to 10 μg *E. coli* LPS (0111:B4, Sigma-Aldrich) or HK *P. melaninogenica* i.t. for killing assays. Killing assays were conducted with 10^3^ *S. aureus* pre-opsonized with 3 % fresh mouse sera for 30 min prior to incubation with 10^5^ neutrophils in Hank’s Balanced Salt Solution (ThermoFisher Scientific) for 1 h at 37 °C under rotation. Percent killing was determined by CFU enumeration following serial dilution of lysed neutrophils, relative to reactions without neutrophils.

### 4.6. Cell culture

The A549 human alveolar epithelial cell line and D562 human pharyngeal epithelial cell line were obtained from the American Type Culture Collection. The CFTR mutant CFBE41o- human bronchial epithelial cell line (CFBE, catalog #SCC151) and the isogenic WT corrected CFBE41o- cell line (WTBE, catalog #SCC158) were obtained from Sigma-Aldrich. Cells were maintained in Ham’s F-12 K (Kaighn’s) medium with 10 % fetal bovine serum (FBS, from CPS Serum, Parkville, MO) and 1 x penicillin-streptomycin (Life Technologies Corporation, Carlsbad, CA). For CFBE and WTBE cells, culture flasks were coated in a 5 mg fibronectin (Sigma-Aldrich, catalog #F2006–5 mg), 50 mg BSA Fraction V (MilliporeSigma, Rockville, MD catalog #126,575), and 15 mg Collagen type I (Sigma-Aldrich, catalog #5006–15 mg) dissolved in 500 mL Minimum Essential Medium (MEM, ThermoFisher Scientific).

Cultured epithelial cells were seeded at a concentration of 2.5 × 10^5^ cells per well 72 h prior to experimental exposures, with multiplicity of infection (MOI) determined based on plated cell density. Cells were exposed to heat-killed *P. melaninogenica* at MOI 0.1, 1, or 10 for 24 h prior to supernatant collection unless otherwise specified. For TLR2 stimulation, cultures were treated with the TLR2 agonist Pam3SK4 at a concentration of 10 ng/mL for 24 h (InvivoGen, San Diego, CA). To block TLR2 signaling, cells were treated with anti-human TLR2 antagonist antibodies (clone TL2.1, Invitrogen, ThermoFisher Scientific, catalog #14–9922–82 and clone 383,936, R&D Systems, Minneapolis, MN, catalog #MAB2616) or isotype control antibodies (IgG2b, clone LTF-2, Bio X Cell #BE0090 and IgG2a, clone C1.18.4, Bio X Cell #BE0089) using a concentration of 1 μg/mL at the time of bacterial exposure. For SCFAs, cells were treated with butyrate or propionate (Sigma-Aldrich) at a concentration of 2.5 mM or 25 mM for 24 h. For CFTR modulators, cells were treated with 0.3 μM elexacaftor (VX-445) (Selleck Chemical LLC, Houston, TX, catalog #S8851) and 0.2 μM tezacaftor (VX-661) (Selleck Chemical LLC, catalog #S7059) at the time of bacterial exposures, with cells treated for 24 h.

### 4.7. Cytokine and chemokine analysis

Cytokines and chemokines were quantified using enzyme-linked immunosorbent assays (ELISA) kits per manufacturer’s instructions (BD OptEIA^™^ Human IL-6 catalog #555,220, BD OptEIA^™^ Human IL-8 catalog #555,244, BD Biosciences and Human CXCL2, R&D Systems). Cell supernatants were collected following pelleting samples at 500 x g for 5 min. Samples were measured using an Agilent BioTek Synergy HTX Multi-Mode Microplate Reader (Agilent BioTek, Winooski, VT).

### 4.8. Epithelial adherence assay

Cells plated for 72 h were exposed to *P. melaninogenica* treatments at the indicated MOI (as above) followed by live *S. aureus* infection at MOI = 0.1 for 1 h. Cells were washed 3x with 1 x PBS prior to treatment with trypsin-EDTA for 30 min at 37 °C followed by the addition of 300 μL of Milli-Q water to lyse all epithelial cells. *S. aureus* burdens were enumerated following serial dilution on mannitol salt agar plates. Percent adherence was calculated relative to cell-free media controls.

### 4.9. RNA-sequencing

CFBE41o- and CFBE41o- WT cells were exposed to heat-killed *P. melaninogenica* or media alone for 24 h. Triplicate wells from each condition were harvested using lysis buffer and RNA was extracted from cell lysates using an RNA extraction kit (Qiagen, RNeasy kit). RNA sequencing was performed by the University of Colorado Genomics Shared Resource core (RRID: SCR_021984). Sequencing libraries were prepared using Nugen Universal Plus mRNA kit and sequenced with an Illumina NovaSEQ X (2 × 150 bp) at a sequencing depth of 37.69 million (± SD of 8.59 million) read pairs.

### 4.10. RNA sequencing analysis

FASTQ files were obtained from the Genomics Shared Resource core. Light trimming and adapter content removal was performed using BBduk (v38.5) [70]. Salmon (v1.4.0) [71] was used to quantify transcripts against a decoy-aware transcriptome index of Gencode H37 (GRCh38) [72]. Transcript counts were summarized at the gene level using Tximport (v1.34.0) [73] followed by differential expression using DESeq2 (v1.46.0) [74]. Overrepresentation analysis (ORA) of Gene Ontology terms was performed using ClusterProfiler (v4.16.0) [75] and the GO database of *org.Hs.eg.db* (v3.21.0) [76]. The input for ORA included differentially expressed genes with FDR-adjusted p value *<*0.05 and ∣log2foldchange∣ *>*1. Log fold change shrinkage was calculated using the “apeglm” algorithm [77]. Shrunken values were used for gene rankings to determine top up and down regulated genes for heatmap visualization. Heatmaps were made using *pheatmap* [78] using RLD-transformed values, scaled by row. Module scoring of individual samples was performed using a modified version [79] of the *AddModuleScore* function in Seurat [80]. Gene lists for module scores are available in Supplemental Table 3. Gene lists for the module scoring and heat maps were derived directly from the indicated GO terms, when listed, or were manually compiled from several GO terms representing differentially expressed genes within the dataset, organized by signaling pathway.

Of note, one sample from the untreated WTBE group was excluded due to significant divergence from other samples in this group. The remaining two samples in the WTBE group displayed closely correlated gene expression patterns (Supplemental Figure 4C). In the remaining untreated WTBE samples, expression of several top differentially enriched genes previously reported for untreated CFBE41o- WT cells versus CFBE41o- CFTR mutant cells [35] were similarly enriched in our dataset for WTBE versus CFBE cells including *CFTR* (Supplemental Figure 4B), *SOX11, MT1E*, and *NGFR* (Supplemental Figure 5).

### 4.11. Statistical analysis

All graphing and statistical analysis was performed on GraphPad Prism (version 10, GraphPad Software LLC, San Diego, CA). Data were tested for normality using the Shapiro-Wilk test. For normal distributions, analysis of variance (ANOVA) and student’s *t*-test was utilized where specified. Mann-Whitney U tests were used for data with non-Gaussian distributions. *P*-values of *<*0.05 were considered significant, with values indicated in Figure legends.

### 4.12. Study approval

All studies were approved by the Institutional Biosafety Committee (protocol #1418) and the Animal Care and Use Committee of the University of Colorado School of Medicine (protocol #927).

## Supplementary Material

Supplementary Table 1

Supplementary Information

Supplementary Table 3

Supplementary Table 4

Supplementary material associated with this article can be found, in the online version, at doi:10.1016/j.jcf.2025.11.002.

## Data Availability

All data supporting the findings of this study, with the exception of RNA sequencing data, are available within the paper and its supplementary files. Transcriptomic data (both raw and processed) can be accessed in NCBI Gene Expression Omnibus (GEO) under accession number GSE305398. All code used for RNA-seq analysis is available through GitHub at https://github.com/eric-d-larson/Clark_CTFR under a GNU General Public License.
