# Supplementary material for: Functional CFTR may be required for Prevotella melaninogenica regulation of epithelial cell defense against Staphylococcus aureus
Source: J Cyst Fibros. Author manuscript; Available in PMC 2026 Jan 22. (PMC12821323; doi:10.1016/j.jcf.2025.11.002)
Supplement: Supplementary Information [file NIHMS2129011-supplement-Supplementary_Information.pdf]

SUPPORTING INFORMATION

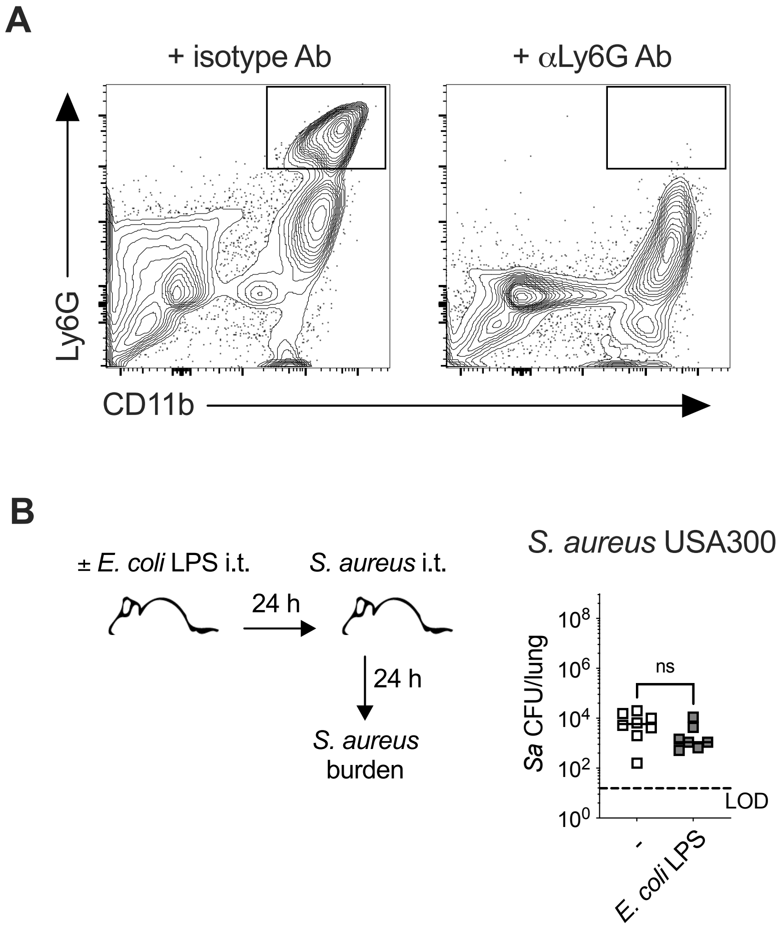

Supplemental Figure 1. **Neutrophil depletion and lack of protection against *S. aureus* following exposure to *E. coli* LPS A** Representative flow cytometry plot of neutrophils (Ly6G$^+$CD11b$^+$ cells from live SiglecF$^-$CD45$^+$ gated cells) detected in lung tissue from mice pre-treated with anti-Ly6G antibody or isotype control antibody (200 μg/mouse) 24 hours prior to live *P. melaninogenica* exposure (10^7 CFU/mouse i.t) followed by challenge with *S. aureus* USA300 (5 x 10^8 CFU/mouse i.t.). **B** Burdens of *S. aureus* USA300 at 24 hours post-infection in mice with or without pre-exposure to *E. coli* LPS (10 μg/mouse i.t.), (n = 7-8 mice/group). Data pooled from two independent experiments, displayed as mean ± SEM.

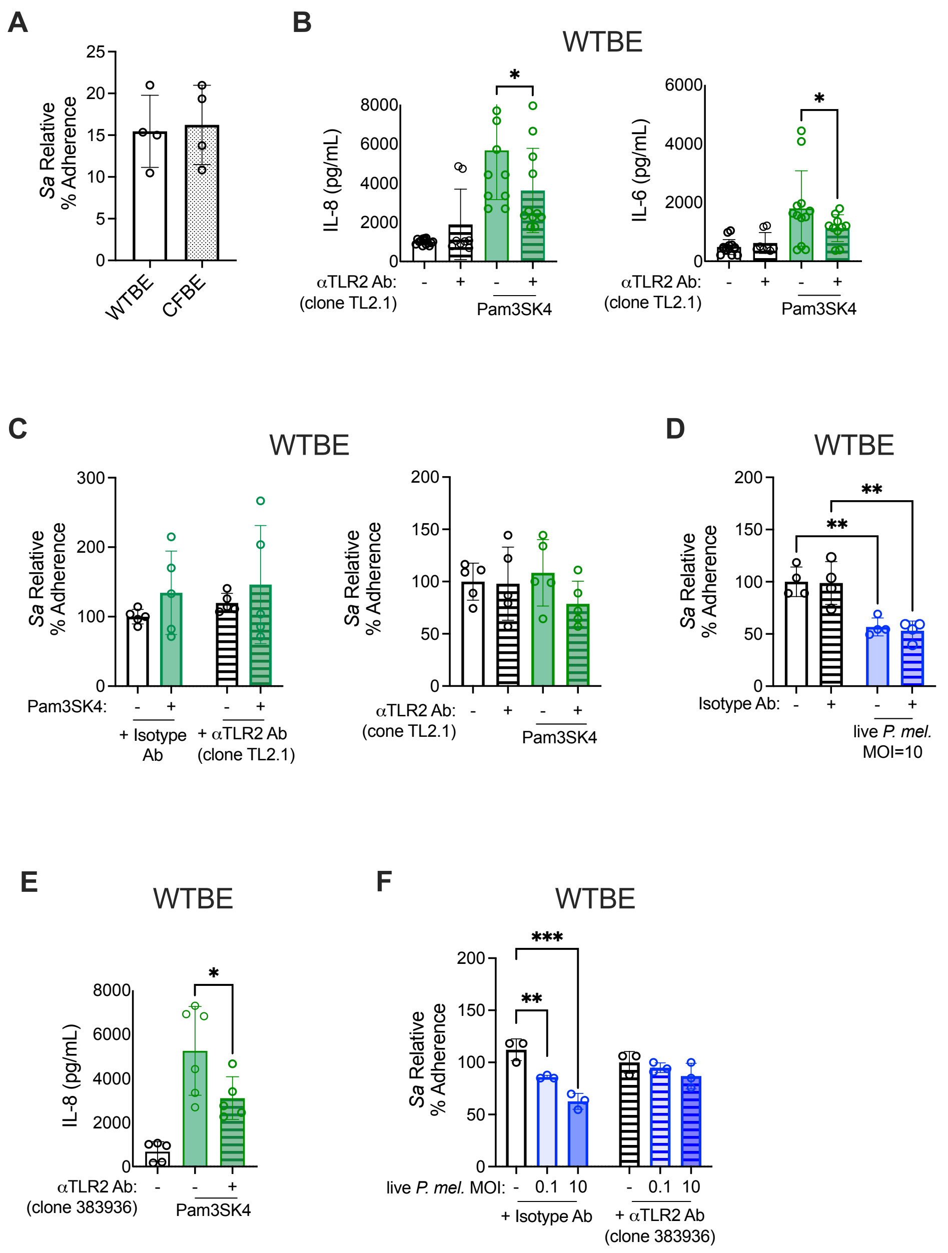

Supplemental Figure 2. **Effects of anti-TLR2 antibody on cytokine production and *S. aureus* adherence. A** *S. aureus* adherence to WTBE and CFTR mutant (CFBE) cells. **B** Supernatant IL-8 and IL-6 detected in WTBE cells 24 hours post-exposure to the TLR2 agonist (Pam3SK4) with or without anti-TLR2 antibody (clone TL2.1). **C** *S. aureus* adherence to WTBE cells pre-exposed to Pam3SK4 with or without anti-TLR2

antibody (clone TL2.1) or isotype control antibody. **D** *S. aureus* adherence to WTBE cells pre-exposed to live *P. melaninogenica* with or without isotype control antibody. **E** Supernatant IL-8 detected in WTBE cells 24 hours post-exposure to Pam3SK4 with anti-TLR2 antibody (clone 38936) or isotype control antibody. **F** *S. aureus* adherence to WTBE cells pre-exposed to live *P. melaninogenica* with anti-TLR2 antibody (clone 38936) or isotype control antibody. Data pooled from two independent experiments, displayed as mean ± SEM. ***p*<.01, ****p*<.001, one-way ANOVA with Dunnett's *post-hoc* analysis.

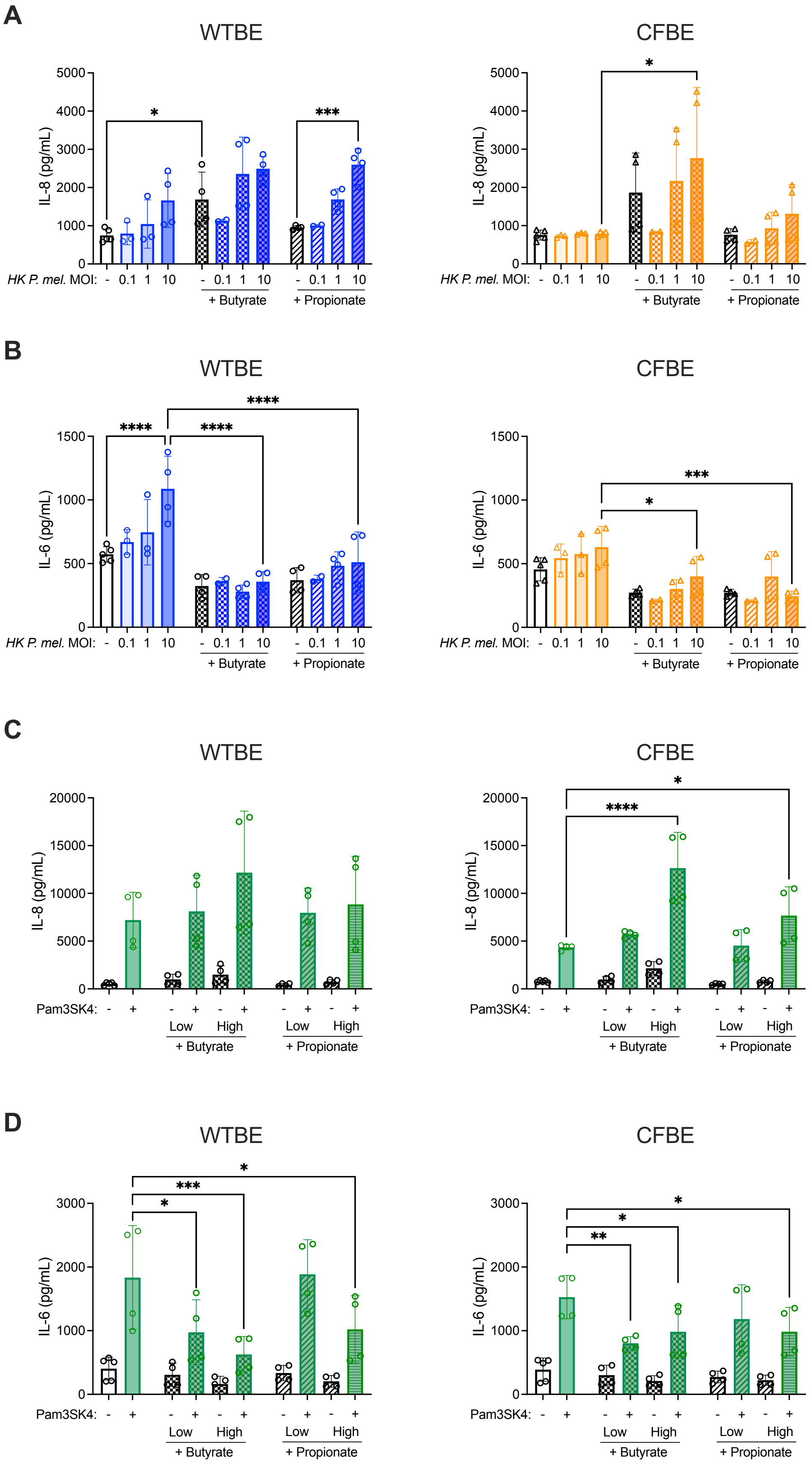
A
WTBE
IL-8 (pg/mL)
5000
4000
3000
2000
1000
0
*
***
HK P. mel. MOI: - 0.1 1 10 - 0.1 1 10 - 0.1 1 10
+ Butyrate
+ Propionate
CFBE
IL-8 (pg/mL)
*
HK P. mel. MOI: - 0.1 1 10 - 0.1 1 10 - 0.1 1 10
+ Butyrate
+ Propionate
B
WTBE
IL-6 (pg/mL)
1500
1000
500
0
****
****
****
HK P. mel. MOI: - 0.1 1 10 - 0.1 1 10 - 0.1 1 10
+ Butyrate
+ Propionate
CFBE
IL-6 (pg/mL)
***
*
HK P. mel. MOI: - 0.1 1 10 - 0.1 1 10 - 0.1 1 10
+ Butyrate
+ Propionate
C
WTBE
IL-8 (pg/mL)
20000
15000
10000
5000
0
Pam3SK4: - + - + - + - + - + - +
Low High Low High
+ Butyrate
+ Propionate
CFBE
IL-8 (pg/mL)
*
****
Pam3SK4: - + - + - + - + - + - +
Low High Low High
+ Butyrate
+ Propionate
D
WTBE
IL-6 (pg/mL)
3000
2000
1000
0
*
***
*
Pam3SK4: - + - + - + - + - + - +
Low High Low High
+ Butyrate
+ Propionate
CFBE
IL-6 (pg/mL)
*
*
**
Pam3SK4: - + - + - + - + - + - +
Low High Low High
+ Butyrate
+ Propionate

E

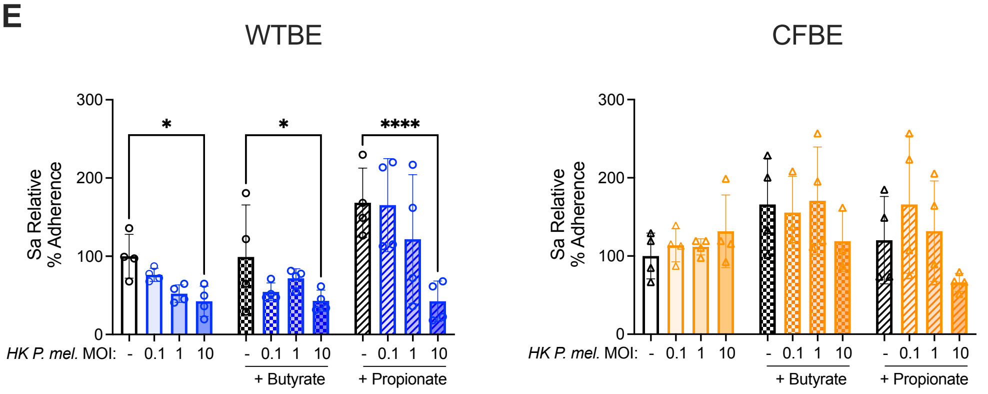

Supplemental 3. SCFAs modulate *P. melaninogenica* and Pam3SK4 induced cytokine production. A-B Supernatant IL-8 (A) and IL-6 (B) detected in WTBE and CFTR mutant (CFBE) cells exposed to heat-killed *P. melaninogenica* with or without 24 mM of butyrate or propionate for 24 hours. **C-D** Supernatant IL-8 (C) and IL-6 (D) detected in WTBE and CFBE cells exposed to Pam3SK4 with or without 2.5 mM (low) or 24 mM (high) of butyrate or propionate for 24 hours. **E** *S. aureus* relative adherence to WTBE and CFBE cells following 24-hour pre-exposure to heat-killed *P. melaninogenica* with or without 24 mM of butyrate or propionate. Data pooled from two to three independent experiments displayed as mean ± SEM. *p<.05, ***p<.001, ****p<.0001, one-way ANOVA with Sidak's *post-hoc* analysis.

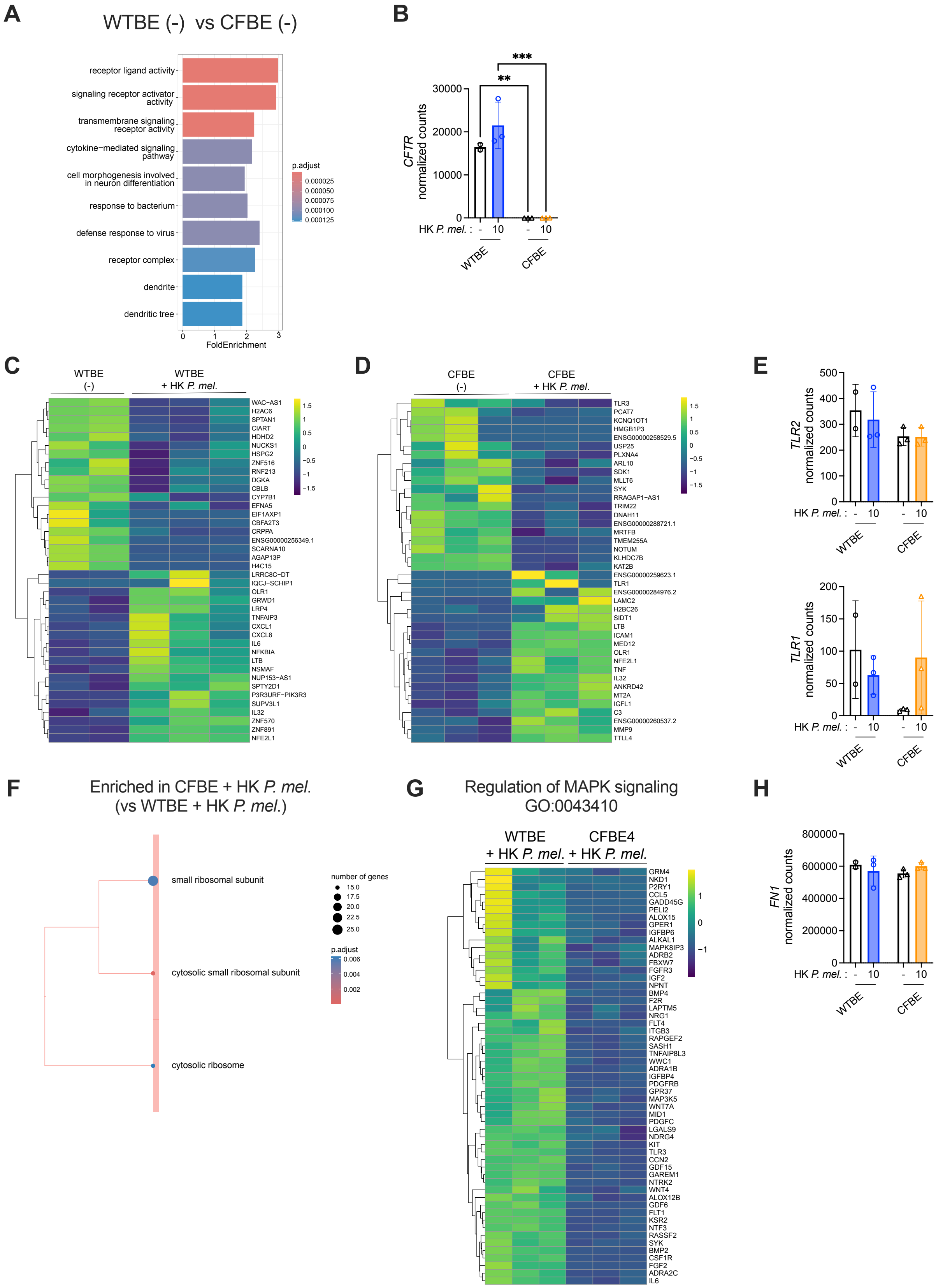
A
WTBE (-) vs CFBE (-)
receptor ligand activity
signaling receptor activator activity
transmembrane signaling receptor activity
cytokine−mediated signaling pathway
cell morphogenesis involved in neuron differentiation
response to bacterium
defense response to virus
receptor complex
dendrite
dendritic tree
FoldEnrichment
p.adjust
0.000025
0.000050
0.000075
0.000100
0.000125
B
CFTR normalized counts
30000
20000
10000
0
***
**
HK P. mel. :
WTBE
CFBE
C
WTBE (-)
WTBE + HK P. mel.
WAC−AS1
H2AC6
SPTAN1
CIART
HDHD2
NUCKS1
HSPG2
ZNF516
RNF213
DGKA
CBLB
CYP7B1
EFNA5
EIF1AXP1
CBFA2T3
CRPPA
ENSG00000256349.1
SCARNA10
AGAP13P
H4C15
LRRC8C−DT
IQCJ−SCHIP1
OLR1
GRWD1
LRP4
TNFAIP3
CXCL1
CXCL8
IL6
NFKBIA
LTB
NSMAF
NUP153−AS1
SPTY2D1
P3R3URF−PIK3R3
SUPV3L1
IL32
ZNF570
ZNF891
NFE2L1
D
CFBE (-)
CFBE + HK P. mel.
TLR3
PCAT7
KCNQ1OT1
HMGB1P3
ENSG00000258529.5
USP25
PLXNA4
ARL10
SDK1
MLLT6
SYK
RRAGAP1−AS1
TRIM22
DNAH11
ENSG00000288721.1
MRTFB
TMEM255A
NOTUM
KLHDC7B
KAT2B
ENSG00000259623.1
TLR1
ENSG00000284976.2
LAMC2
H2BC26
SIDT1
LTB
ICAM1
MED12
OLR1
NFE2L1
TNF
IL32
ANKRD42
MT2A
IGFL1
C3
ENSG00000260537.2
MMP9
TTLL4
E
TLR2 normalized counts
TLR1 normalized counts
HK P. mel. :
WTBE
CFBE
F
Enriched in CFBE + HK P. mel. (vs WTBE + HK P. mel.)
small ribosomal subunit
cytosolic small ribosomal subunit
cytosolic ribosome
number of genes
15.0
17.5
20.0
22.5
25.0
p.adjust
0.006
0.004
0.002
G
Regulation of MAPK signaling GO:0043410
WTBE + HK P. mel.
CFBE4 + HK P. mel.
GRM4
NKD1
P2RY1
CCL5
GADD45G
PELI2
ALOX15
GPER1
IGFBP6
ALKAL1
MAPK8IP3
ADRB2
FBXW7
FGFR3
IGF2
NPNT
BMP4
F2R
LAPTM5
NRG1
FLT4
ITGB3
RAPGEF2
SASH1
TNFAIP8L3
WWC1
ADRA1B
IGFBP4
PDGFRB
GPR37
MAP3K5
WNT7A
MID1
PDGFC
LGALS9
NDRG4
KIT
TLR3
CCN2
GDF15
GAREM1
NTRK2
WNT4
ALOX12B
GDF6
FLT1
KSR2
NTF3
RASSF2
SYK
BMP2
CSF1R
FGF2
ADRA2C
IL6
H
FN1 normalized counts
800000
600000
400000
200000
0
HK P. mel. :
WTBE
CFBE

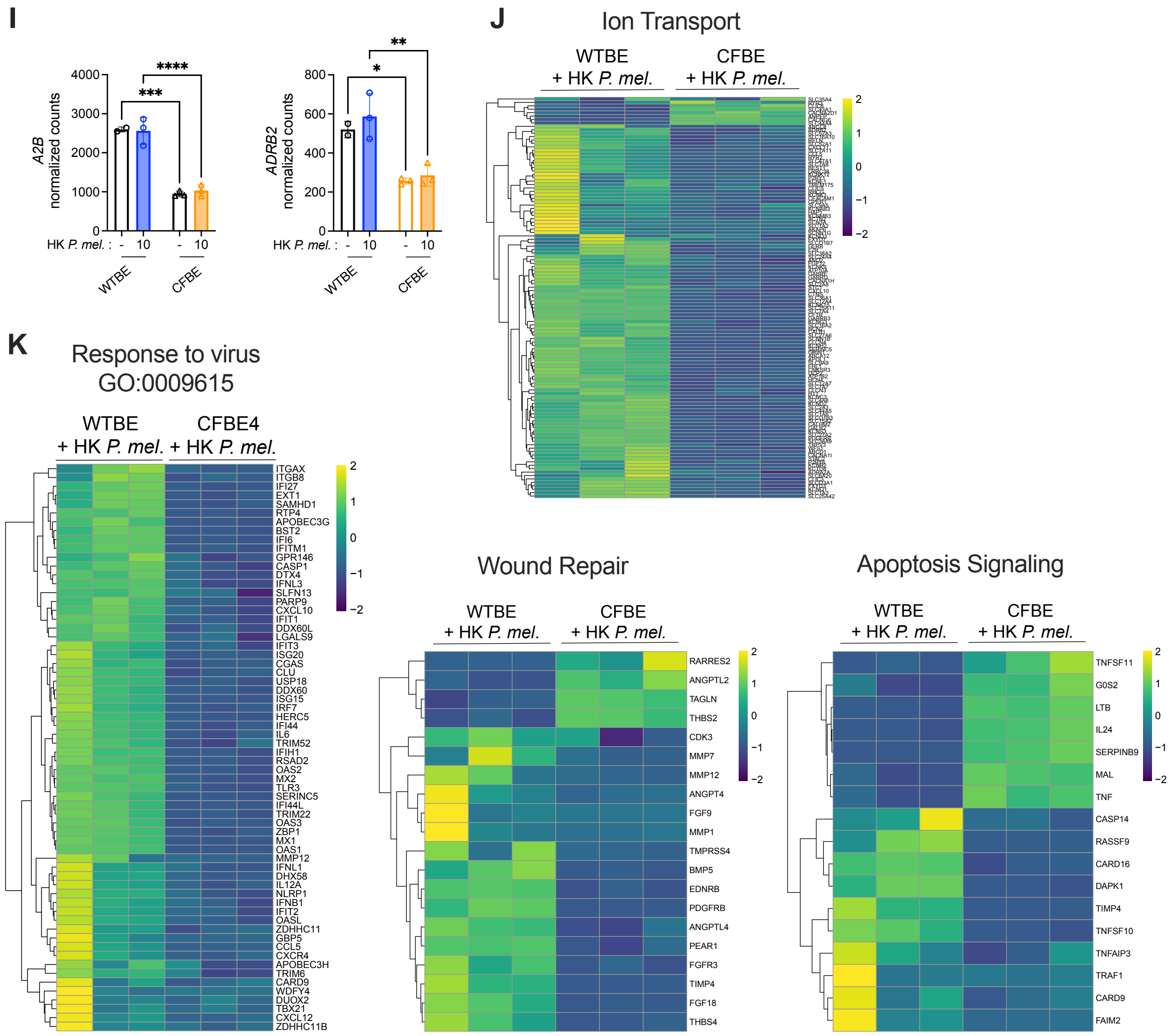

Supplemental Figure 4. Differential gene expression patterns between CFTR functional and mutant cells. A Fold enrichment of the top ten significant differentially expressed gene ontology terms in untreated WTBE cells compared to untreated CFTR mutant (CFBE) cells. **B** Normalized gene counts for *CFTR* in WTBE cell and CFBE cells with or without exposure to heat-killed *P. melaninogenica.* **C-D** Heat maps of the top differentially expressed genes in untreated WTBE cells with or without exposure to heat-killed *P. melaninogenica* (C) or CFBE cells with or without exposure to heat-killed *P. melaninogenica* (D). **E** Normalized gene counts for *TLR2* and *TLR1* in WTBE and CFBE

cells with or without exposure to heat-killed *P. melaninogenica.* **F** Tree plot of top significant gene ontology terms in CFBE cells exposed to *P. melaninogenica* compared to WTBE cells exposed to *P. melaninogenica*. **G** Heat map of all significantly differentially enriched genes from GO term for regulation of MAPK signaling (GO:0043410) in CFBE cells exposed to *P. melaninogenica* compared to WTBE cells exposed to *P. melaninogenica*. **H-I** Normalized gene counts for *FN1* (H), *A2B* and *ADRB2* (I) in WTBE and CFBE cells with or without exposure to heat-killed *P. melaninogenica.* **J** Heat maps of genes related to Ion Transport for WTBE cells exposed to *P. melaninogenica* compared to CFBE cells exposed to *P. melaninogenica*. **K** Heat maps of all significant differentially enriched genes from GO term for response to virus (GO:0009615), genes related to wound repair, and genes related to apoptosis signaling in CFBE cells exposed to *P. melaninogenica* compared to WTBE cells exposed to *P. melaninogenica*. Gene count data displayed as individual points plus mean ± SEM. *$p<.05$, ***$p<.001$, ****$p<.0001$, one-way ANOVA with Sidak's *post-hoc* analysis.

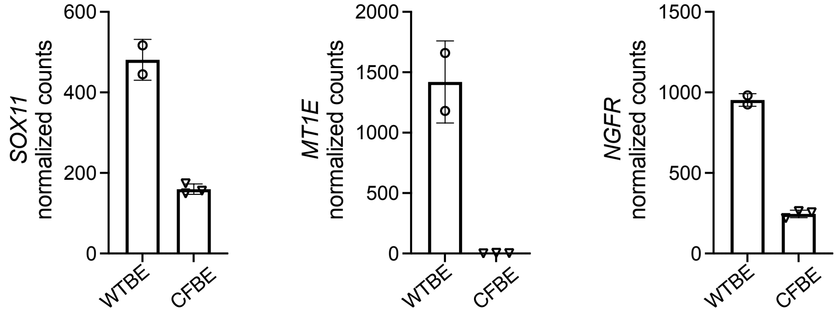

Supplemental Figure 5. Select gene expression in WTBE versus CFBE cells. Normalized expression counts for *SOX11, MT1E,* and *NGFR* in untreated WTBE and CFBE cells*.* Gene count data displayed as individual points plus mean ± SEM.

Supplemental Tables

Supplemental Table 1. Differential expression analysis. DESeq2 output of tests performed on the RNA-sequencing data.

Supplemental Table 2. Gene ontology over representation analysis. Filtered output of *ClusterProfiler* over-representation analysis using GO:BP, GO:MF, and GO:CC

databases. Only GO terms with qvalue < 0.05 are included. “Enriched Group” column denotes which group GO term was identified as over represented for each comparison.

Supplemental Table 3. Curated gene lists for module score analysis and heat maps. Gene lists used in the module score analysis depicted in Figure 5 and heat maps in Supplemental Figure 4.
